# Supplementary material for: Epistatic interactions between killer immunoglobulin-like receptors and human leukocyte antigen ligands are associated with ankylosing spondylitis
Source: PLoS Genet. 2020 Aug 17;16(8):e1008906. doi: 10.1371/journal.pgen.1008906 (PMC7451988; doi:10.1371/journal.pgen.1008906)
Supplement: S1 Table — ‘KIR Haplotype’ corresponds to the KIRhaplotype number reported by KIR*IMP based on gene content, ‘AvsB’ provides the A/B haplotype classification. Dosage of each gene is indicated by colour (white = 0, pale green = 1, dark green = 2). KIR2DS4D = KIR2DS4 deletion allele, KIR2DS4W = KIR2DS4 wild-type, KIR2DS4T = KIR2DS4 TOTAL (sum of wild-type and deletion alleles). Isoforms of KIR3DL1 with variable inclusion of exons 4 and 9 are indicated by KIR3DL1ex4 and KIR3Dl1ex9 respectively. (DOCX) [file pgen.1008906.s001.docx]

| ***KIR Haplotype*** | ***AvsB*** | ***KIR3DL3*** | ***KIR2DS2*** | ***KIR2DL2*** | ***KIR2DL3*** | ***KIR2DP1*** | ***KIR2DL1*** | ***KIR3DP1*** | ***KIR2DL4*** | ***KIR3DL1ex4*** | ***KIR3DL1ex9*** | ***KIR3DS1*** | ***KIR2DL5*** | ***KIR2DS3*** | ***KIR2DS5*** | ***KIR2DS1*** | ***KIR2DS4T*** | ***KIR2DS4W*** | ***KIR2DS4D*** | ***KIR3DL2*** |
| --- | --- | --- | --- | --- | --- | --- | --- | --- | --- | --- | --- | --- | --- | --- | --- | --- | --- | --- | --- | --- |
| **1** | **A** |  |  |  |  |  |  |  |  |  |  |  |  |  |  |  |  |  |  |  |
| **2** | **A** |  |  |  |  |  |  |  |  |  |  |  |  |  |  |  |  |  |  |  |
| **3** | **B** |  |  |  |  |  |  |  |  |  |  |  |  |  |  |  |  |  |  |  |
| **4** | **B** |  |  |  |  |  |  |  |  |  |  |  |  |  |  |  |  |  |  |  |
| **5** | **B** |  |  |  |  |  |  |  |  |  |  |  |  |  |  |  |  |  |  |  |
| **6** | **B** |  |  |  |  |  |  |  |  |  |  |  |  |  |  |  |  |  |  |  |
| **7** | **B** |  |  |  |  |  |  |  |  |  |  |  |  |  |  |  |  |  |  |  |
| **8** | **B** |  |  |  |  |  |  |  |  |  |  |  |  |  |  |  |  |  |  |  |
| **9** | **B** |  |  |  |  |  |  |  |  |  |  |  |  |  |  |  |  |  |  |  |
| **10** | **B** |  |  |  |  |  |  |  |  |  |  |  |  |  |  |  |  |  |  |  |
| **11** | **B** |  |  |  |  |  |  |  |  |  |  |  |  |  |  |  |  |  |  |  |
| **12** | **B** |  |  |  |  |  |  |  |  |  |  |  |  |  |  |  |  |  |  |  |
| **13** | **B** |  |  |  |  |  |  |  |  |  |  |  |  |  |  |  |  |  |  |  |
| **14** | **B** |  |  |  |  |  |  |  |  |  |  |  |  |  |  |  |  |  |  |  |
| **15** | **B** |  |  |  |  |  |  |  |  |  |  |  |  |  |  |  |  |  |  |  |
| **16** | **B** |  |  |  |  |  |  |  |  |  |  |  |  |  |  |  |  |  |  |  |
| **17** | **B** |  |  |  |  |  |  |  |  |  |  |  |  |  |  |  |  |  |  |  |
| **18** | **B** |  |  |  |  |  |  |  |  |  |  |  |  |  |  |  |  |  |  |  |
| **19** | **B** |  |  |  |  |  |  |  |  |  |  |  |  |  |  |  |  |  |  |  |
| **20** | **B** |  |  |  |  |  |  |  |  |  |  |  |  |  |  |  |  |  |  |  |
| **21** | **B** |  |  |  |  |  |  |  |  |  |  |  |  |  |  |  |  |  |  |  |
| **22** | **B** |  |  |  |  |  |  |  |  |  |  |  |  |  |  |  |  |  |  |  |
| **23** | **B** |  |  |  |  |  |  |  |  |  |  |  |  |  |  |  |  |  |  |  |
| **24** | **B** |  |  |  |  |  |  |  |  |  |  |  |  |  |  |  |  |  |  |  |
| **25** | **B** |  |  |  |  |  |  |  |  |  |  |  |  |  |  |  |  |  |  |  |
| **27** | **A** |  |  |  |  |  |  |  |  |  |  |  |  |  |  |  |  |  |  |  |
| **28** | **B** |  |  |  |  |  |  |  |  |  |  |  |  |  |  |  |  |  |  |  |
| **29** | **B** |  |  |  |  |  |  |  |  |  |  |  |  |  |  |  |  |  |  |  |
| **30** | **B** |  |  |  |  |  |  |  |  |  |  |  |  |  |  |  |  |  |  |  |
| **31** | **B** |  |  |  |  |  |  |  |  |  |  |  |  |  |  |  |  |  |  |  |
| **33** | **B** |  |  |  |  |  |  |  |  |  |  |  |  |  |  |  |  |  |  |  |
| **34** | **A** |  |  |  |  |  |  |  |  |  |  |  |  |  |  |  |  |  |  |  |
| **36** | **B** |  |  |  |  |  |  |  |  |  |  |  |  |  |  |  |  |  |  |  |
| **38** | **B** |  |  |  |  |  |  |  |  |  |  |  |  |  |  |  |  |  |  |  |
| **40** | **B** |  |  |  |  |  |  |  |  |  |  |  |  |  |  |  |  |  |  |  |
| **41** | **B** |  |  |  |  |  |  |  |  |  |  |  |  |  |  |  |  |  |  |  |
| **42** | **B** |  |  |  |  |  |  |  |  |  |  |  |  |  |  |  |  |  |  |  |
| **44** | **B** |  |  |  |  |  |  |  |  |  |  |  |  |  |  |  |  |  |  |  |
| **45** | **B** |  |  |  |  |  |  |  |  |  |  |  |  |  |  |  |  |  |  |  |
| **46** | **B** |  |  |  |  |  |  |  |  |  |  |  |  |  |  |  |  |  |  |  |
| **48** | **A** |  |  |  |  |  |  |  |  |  |  |  |  |  |  |  |  |  |  |  |
| **50** | **B** |  |  |  |  |  |  |  |  |  |  |  |  |  |  |  |  |  |  |  |
| **52** | **B** |  |  |  |  |  |  |  |  |  |  |  |  |  |  |  |  |  |  |  |
| **53** | **B** |  |  |  |  |  |  |  |  |  |  |  |  |  |  |  |  |  |  |  |
| **55** | **A** |  |  |  |  |  |  |  |  |  |  |  |  |  |  |  |  |  |  |  |
| **56** | **B** |  |  |  |  |  |  |  |  |  |  |  |  |  |  |  |  |  |  |  |
| **57** | **A** |  |  |  |  |  |  |  |  |  |  |  |  |  |  |  |  |  |  |  |
| **58** | **A** |  |  |  |  |  |  |  |  |  |  |  |  |  |  |  |  |  |  |  |
| **59** | **A** |  |  |  |  |  |  |  |  |  |  |  |  |  |  |  |  |  |  |  |
| **68** | **B** |  |  |  |  |  |  |  |  |  |  |  |  |  |  |  |  |  |  |  |
| **69** | **B** |  |  |  |  |  |  |  |  |  |  |  |  |  |  |  |  |  |  |  |
| ***KIRHaplotype*** | ***AvsB*** | ***KIR3DL3*** | ***KIR2DS2*** | ***KIR2DL2*** | ***KIR2DL3*** | ***KIR2DP1*** | ***KIR2DL1*** | ***KIR3DP1*** | ***KIR2DL4*** | ***KIR3DL1ex4*** | ***KIR3DL1ex9*** | ***KIR3DS1*** | ***KIR2DL5*** | ***KIR2DS3*** | ***KIR2DS5*** | ***KIR2DS1*** | ***KIR2DS4T*** | ***KIR2DS4W*** | ***KIR2DS4D*** | ***KIR3DL2*** |
