# Supplementary material for: Epistatic interactions between killer immunoglobulin-like receptors and human leukocyte antigen ligands are associated with ankylosing spondylitis
Source: PLoS Genet. 2020 Aug 17;16(8):e1008906. doi: 10.1371/journal.pgen.1008906 (PMC7451988; doi:10.1371/journal.pgen.1008906)
Supplement: S2 Table — Common haplotypes exceeding 1% frequency in the published dataset are shaded in grey. (DOCX) [file pgen.1008906.s002.docx]

|  | **Haplotype Frequency** | | |
| --- | --- | --- | --- |
| **KIR Haplotype** | **Frequency in IGAS**  **test cohort (%)**  **(40,642 chromosomes)** | **Frequency in UKB**  **replication cohort (%)**  **(36,682 chromosomes)** | **Frequency in**  **Jiang et. al. (%)**  **(2,999 chromosomes)** |
| **A1** | 47.04 | 47.10 | **42.21** |
| **A2** | 13.08 | 13.53 | **12.97** |
| **B3** | 10.82 | 11.42 | **10.90** |
| **B4** | 6.61 | 4.84 | **7.24** |
| **B5** | 6.41 | 5.74 | **5.54** |
| **B6** | 3.39 | 2.07 | **5.10** |
| **B9** | 1.87 | 1.71 | **2.10** |
| **B8** | 0.79 | 0.68 | **2.13** |
| **B10** | 1.54 | 0.78 | **1.83** |
| **B7** | 0.76 | 0.44 | **2.57** |
| **B51** | 1.01 | 3.15 | **0.03** |
| **B11** | 1.06 | 0.98 | **1.43** |
| **B25** | 0.40 | 0.43 | **0.10** |
| **B24** | 0.16 | 0.11 | **0.10** |
| **B44** | 0.11 | 0.05 | **0.03** |
| **B17** | 0.06 | 0.02 | **0.27** |
| **B14** | 0.03 | 0.17 | **0.43** |
| **A27** | 0.02 | 0.003 | **0.10** |
| **B13** | 0.01 | 0.01 | **0.67** |
