# Supplementary material for: Epistatic interactions between killer immunoglobulin-like receptors and human leukocyte antigen ligands are associated with ankylosing spondylitis
Source: PLoS Genet. 2020 Aug 17;16(8):e1008906. doi: 10.1371/journal.pgen.1008906 (PMC7451988; doi:10.1371/journal.pgen.1008906)
Supplement: S3 Table — Proportions are calculated across all haplotypes from HLA-B*27+ controls in the study cohort (2n = 198). High and low frequency alleles are defined as in Boudreau et al. (DOCX) [file pgen.1008906.s003.docx]

| KIR3DL1 Allele Group | High Frequency Alleles (>1%) | Low Frequency Alleles (<1%) | HLA-B*27+ CO % (Count) | Boudreau *et al.*  % |
| --- | --- | --- | --- | --- |
| KIR3DL1 High-1 | KIR3DL1*001, *016 | KIR3DL1*026, *027, *043, *052, *059, *060, *061, *064, *065, *067, *075 | 21.7 (43/198) | 19.2 |
| KIR3DL1 High-2 | KIR3DL1*002, *015, *008, *009, *020 | KIR3DL1*006, *017, *018, *022, *023, *024N, *025, *028, *029, *030, *031, *034, *035, *038, *042, *051, *054, *057, *062, *066, *074, *076, *077 | 24.7 (49/198) | 26.6 |
| KIR3DL1 Low-1 | KIR3DL1*005 | KIR3DL1*041, *044, *053 | 14.1 (28/198) | 14.1 |
| KIR3DL1 Low-2 | KIR3DL1*007 | KIR3DL1*032, *033, *068 | 3.5 (7/198) | 2.7 |
| KIR3DL1 Null | KIR3DL1*004 | KIR3DL1*019, *021, *036, *037, *039, *040, *056, *063, *072 | 16.2 (32/198) | 16.9 |
| KIR3DS1 | KIR3DS1*013 | KIR3DS1*010, *011, *012, *014, *045, *046, *047, *048, *049N, *050, *055, *058 | 19.7 (39/198) | 19.8 |
