# Supplementary material for: Epistatic interactions between killer immunoglobulin-like receptors and human leukocyte antigen ligands are associated with ankylosing spondylitis
Source: PLoS Genet. 2020 Aug 17;16(8):e1008906. doi: 10.1371/journal.pgen.1008906 (PMC7451988; doi:10.1371/journal.pgen.1008906)
Supplement: S4 Table — Concordance was calculated on an individual rather than a haplotype basis as phasing was undetermined for laboratory typed samples. Percent concordance is reported either for all individuals (n = 52), or just those with all gene dosages imputed with posterior probability above 0.4 (n = 51) or 0.6 (n = 37). KIR2DS4D = KIR2DS4 deletion allele, KIR2DS4W = KIR2DS4 wild-type. (DOCX) [file pgen.1008906.s004.docx]

|  | **Per-gene posterior probability threshold** | | |
| --- | --- | --- | --- |
| **Gene** | **0 (n=52)** | **0.4 (n=51)** | **0.6 (n=37)** |
| **KIR2DS2** | 100 | 100 | 100 |
| **KIR2DL2** | 100 | 100 | 100 |
| **KIR2DL3** | 100 | 100 | 100 |
| **KIR2DP1** | 88.5 | 90.2 | 100 |
| **KIR2DL1** | 88.5 | 90.2 | 100 |
| **KIR3DP1** | 100 | 100 | 100 |
| **KIR2DL4** | 100 | 100 | 100 |
| **KIR3DS1** | 100 | 100 | 100 |
| **KIR2DS1** | 98 | 98 | 100 |
| **KIR2DS4W** | 96 | 96 | 100 |
| **KIR2DS4D** | 96 | 96 | 100 |
