# Supplementary material for: Epistatic interactions between killer immunoglobulin-like receptors and human leukocyte antigen ligands are associated with ankylosing spondylitis
Source: PLoS Genet. 2020 Aug 17;16(8):e1008906. doi: 10.1371/journal.pgen.1008906 (PMC7451988; doi:10.1371/journal.pgen.1008906)
Supplement: S7 Table — Interactions in blue are those between receptors and HLA subclasses ligands known to biologically interact. Alternate shading indicates groups of KIRs in strong LD that demonstrate statistical interactions with the same HLA subclass ligand. P = P-value denoting significance of the KIR association with AS when assessed in the specified HLA allele group, Int.P = P-value for the KIR-HLA allele interaction term, * = P-values that remain significant (P<0.05) after multiple testing correction, + = dominant inheritance, ++ = recessive inheritance (homozygosity), AS = ankylosing spondylitis, CTRL = control, OR = odds ratio, SE = standard error, NS = not significant, KIR2DS4D = KIR2DS4 deletion allele, KIR2DS4W = KIR2DS4 wild-type. Proportions are the proportion of individuals with the specified KIR genotype in cohorts split by HLA allele carriage. (DOCX) [file pgen.1008906.s007.docx]

|  |  | **TEST COHORT** | | | | | | **REPLICATION COHORT** | | | | | |
| --- | --- | --- | --- | --- | --- | --- | --- | --- | --- | --- | --- | --- | --- |
| **KIR** | **HLA subclass** | **AS Prop. (Count)** | **CO Prop. (Count)** | **OR** | **SE** | **P** | **Int.P** | **AS Prop. (Count)** | **CO Prop. (Count)** | **OR** | **SE** | **P** | **Int.P** |
| 2DS1++ | Bw4A+ | 0.025(60/2360) | 0.042(147/3471) | 0.59 | 0.16 | **6.23x10^-4^*** | **2.30x10^-4^*** | 0.022(21/967) | 0.035(141/4044) | 0.62 | 0.24 | **0.04** | **0.03** |
|  | Bw4A- | 0.04(230/5747) | 0.035(307/8743) | 1.14 | 0.09 | 0.15 |  | 0.038(97/2530) | 0.035(382/10800) | 1.09 | 0.12 | 0.46 |  |
| 3DL1+ | Bw4A+ | 0.975(2302/2360) | 0.958(3324/3471) | 1.77 | 0.16 | **3.12x10^-4^*** | **2.43x10^-4^*** | 0.979(947/967) | 0.965(3903/4044) | 1.71 | 0.24 | **0.03** | **0.02** |
|  | Bw4A- | 0.961(5525/5747) | 0.965(8438/8743) | 0.91 | 0.09 | 0.27 |  | 0.962(2433/2530) | 0.965(10418/10800) | 0.92 | 0.12 | 0.46 |  |
| 2DS4T+ | Bw4A+ | 0.975(2302/2360) | 0.958(3324/3471) | 1.77 | 0.16 | **3.12x10^-4^*** | **2.64x10^-4^*** | 0.978(946/967) | 0.965(3903/4044) | 1.62 | 0.24 | **0.04** | **0.03** |
|  | Bw4A- | 0.961(5525/5747) | 0.965(8437/8743) | 0.91 | 0.09 | 0.29 |  | 0.962(2433/2530) | 0.965(10418/10800) | 0.92 | 0.12 | 0.46 |  |
| 3DS1++ | Bw4A+ | 0.03(70/2360) | 0.049(170/3471) | 0.59 | 0.15 | **2.56x10^-4^*** | **4.64x10^-4^*** | 0.025(24/967) | 0.04(161/4044) | 0.61 | 0.22 | **0.03** | **0.02** |
|  | Bw4A- | 0.044(250/5747) | 0.041(358/8743) | 1.06 | 0.08 | 0.48 |  | 0.043(108/2530) | 0.04(433/10800) | 1.07 | 0.11 | 0.54 |  |
| 2DS5++ | Bw4A+ | 0.014(33/2360) | 0.024(85/3471) | 0.56 | 0.21 | **5.68x10^-3^*** | **8.54x10^-4^*** | 0.01(10/967) | 0.023(95/4044) | 0.44 | 0.34 | **0.01** | **0.01** |
|  | Bw4A- | 0.024(140/5747) | 0.02(172/8743) | 1.24 | 0.12 | 0.07 |  | 0.023(59/2530) | 0.022(235/10800) | 1.07 | 0.15 | 0.64 |  |
| 3DL1++ | C1+ | 0.675(4517/6692) | 0.655(6972/10637) | 1.10 | 0.03 | **5.07x10^-3^*** | **0.02** | 0.676(1984/2936) | 0.67(8663/12938) | 1.03 | 0.04 | 0.53 | 0.51 |
|  | C1- | 0.668(945/1415) | 0.693(1093/1577) | 0.90 | 0.08 | 0.19 |  | 0.688(386/561) | 0.668(1273/1906) | 1.10 | 0.10 | 0.35 |  |
| 2DS4T++ | C1+ | 0.675(4515/6692) | 0.655(6969/10637) | 1.10 | 0.03 | **5.11x10^-3^*** | **0.02** | 0.676(1984/2936) | 0.669(8656/12938) | 1.03 | 0.04 | 0.50 | 0.50 |
|  | C1- | 0.668(945/1415) | 0.692(1092/1577) | 0.90 | 0.08 | 0.20 |  | 0.688(386/561) | 0.667(1271/1906) | 1.11 | 0.10 | 0.33 |  |
| 2DS1+ | C1+ | 0.325(2174/6692) | 0.345(3668/10637) | 0.91 | 0.03 | **4.20x10^-3^** | **0.02** | 0.324(952/2936) | 0.331(4279/12938) | 0.97 | 0.04 | 0.51 | 0.51 |
|  | C1- | 0.333(471/1415) | 0.309(487/1577) | 1.10 | 0.08 | 0.21 |  | 0.312(175/561) | 0.333(634/1906) | 0.91 | 0.10 | 0.34 |  |
| 3DS1+ | C1+ | 0.324(2171/6692) | 0.343(3645/10637) | 0.92 | 0.03 | **8.67x10^-3^** | **0.03** | 0.324(950/2936) | 0.328(4244/12938) | 0.98 | 0.04 | 0.66 | 0.33 |
|  | C1- | 0.329(465/1415) | 0.306(483/1577) | 1.09 | 0.08 | 0.25 |  | 0.305(171/561) | 0.331(631/1906) | 0.88 | 0.10 | 0.23 |  |
| 2DL5+ | C2+ | 0.439(2383/5427) | 0.438(3147/7193) | 1.00 | 0.04 | 0.94 | **0.02** | 0.377(864/2291) | 0.395(3401/8600) | 0.92 | 0.05 | 0.10 | 0.65 |
|  | C2- | 0.432(1159/2680) | 0.467(2346/5021) | 0.86 | 0.05 | **1.65x10^-3^*** |  | 0.383(462/1206) | 0.394(2461/6244) | 0.96 | 0.07 | 0.50 |  |
| 3DS1+ | C2+ | 0.329(1786/5427) | 0.33(2372/7193) | 0.99 | 0.04 | 0.84 | **0.03** | 0.32(733/2291) | 0.33(2839/8600) | 0.95 | 0.05 | 0.33 | 0.71 |
|  | C2- | 0.317(850/2680) | 0.35(1756/5021) | 0.86 | 0.05 | **2.70x10^-3^*** |  | 0.322(388/1206) | 0.326(2036/6244) | 0.98 | 0.07 | 0.78 |  |
| 3DL1++ | C2+ | 0.67(3637/5427) | 0.668(4806/7193) | 1.01 | 0.04 | 0.72 | **0.04** | 0.678(1554/2291) | 0.668(5742/8600) | 1.05 | 0.05 | 0.31 | 0.71 |
|  | C2- | 0.681(1825/2680) | 0.649(3259/5021) | 1.16 | 0.05 | **3.16x10^-3^*** |  | 0.677(816/1206) | 0.672(4194/6244) | 1.02 | 0.07 | 0.75 |  |
| 2DS4T++ | C2+ | 0.67(3636/5427) | 0.668(4804/7193) | 1.01 | 0.04 | 0.71 | **0.04** | 0.678(1554/2291) | 0.667(5737/8600) | 1.06 | 0.05 | 0.29 | 0.71 |
|  | C2- | 0.681(1824/2680) | 0.649(3257/5021) | 1.16 | 0.05 | **3.15x10^-3^*** |  | 0.677(816/1206) | 0.671(4190/6244) | 1.02 | 0.07 | 0.72 |  |
| 2DS1+ | C2+ | 0.33(1790/5427) | 0.333(2392/7193) | 0.98 | 0.04 | 0.66 | **0.04** | 0.322(737/2291) | 0.333(2861/8600) | 0.95 | 0.05 | 0.30 | 0.71 |
|  | C2- | 0.319(855/2680) | 0.351(1763/5021) | 0.86 | 0.05 | **2.96x10^-3^*** |  | 0.323(390/1206) | 0.329(2052/6244) | 0.98 | 0.07 | 0.74 |  |
| 2DS3+ | C2+ | 0.205(1113/5427) | 0.199(1435/7193) | 1.03 | 0.05 | 0.53 | **0.04** | 0.138(317/2291) | 0.147(1260/8600) | 0.93 | 0.07 | 0.31 | 0.30 |
|  | C2- | 0.196(525/2680) | 0.216(1083/5021) | 0.88 | 0.06 | **0.03** |  | 0.148(179/1206) | 0.143(893/6244) | 1.05 | 0.09 | 0.62 |  |
| 2DS5+ | Bw4B(I80)+ | 0.273(367/1345) | 0.25(704/2815) | 1.1 | 0.08 | 0.12 | **0.02** | 0.305(147/482) | 0.265(850/3202) | 1.20 | 0.11 | 0.08 | **0.01** |
|  | Bw4B(I80)- | 0.252(1704/6762) | 0.267(2505/9399) | 0.9 | 0.04 | **0.03** |  | 0.248(747/3015) | 0.267(3111/11642) | 0.90 | 0.05 | **0.03** |  |
| 2DL5+ | Bw4B(I80)+ | 0.469(631/1345) | 0.447(1259/2815) | 1.1 | 0.07 | 0.28 | **0.02** | 0.423(204/482) | 0.394(1261/3202) | 1.12 | 0.10 | 0.26 | **0.05** |
|  | Bw4B(I80)- | 0.43(2911/6762) | 0.45(4234/9399) | 0.9 | 0.03 | **4.67x10^-3^*** |  | 0.372(1122/3015) | 0.395(4601/11642) | 0.91 | 0.04 | **0.02** |  |
| 3DS1+ | Bw4B(I80)+ | 0.346(465/1345) | 0.329(925/2815) | 1.1 | 0.07 | 0.29 | **0.03** | 0.367(177/482) | 0.327(1048/3202) | 1.18 | 0.10 | 0.11 | **0.03** |
|  | Bw4B(I80)- | 0.321(2171/6762) | 0.341(3203/9399) | 0.9 | 0.03 | **5.28x10^-3^*** |  | 0.313(944/3015) | 0.329(3827/11642) | 0.93 | 0.04 | 0.11 |  |
| 2DS1+ | Bw4B(I80)+ | 0.346(466/1345) | 0.333(938/2815) | 1.1 | 0.07 | 0.42 | **0.05** | 0.367(177/482) | 0.331(1059/3202) | 1.16 | 0.10 | 0.14 | **0.04** |
|  | Bw4B(I80)- | 0.322(2179/6762) | 0.342(3217/9399) | 0.9 | 0.03 | **4.54x10^-3^*** |  | 0.315(950/3015) | 0.331(3854/11642) | 0.93 | 0.04 | 0.10 |  |
| 3DL1++ | Bw4B(I80)+ | 0.654(880/1345) | 0.667(1879/2815) | 0.9 | 0.07 | 0.42 | 0.05 | 0.633(305/482) | 0.67(2145/3202) | 0.86 | 0.10 | 0.14 | **0.04** |
|  | Bw4B(I80)- | 0.678(4582/6762) | 0.658(6186/9399) | 1.1 | 0.03 | **5.80x10^-3^*** |  | 0.685(2065/3015) | 0.669(7791/11642) | 1.07 | 0.04 | 0.11 |  |
| 2DS3+ | Bw4B+ | 0.205(1559/7619) | 0.206(1518/7365) | 1 | 0.04 | 0.80 | **0.03** | 0.144(471/3276) | 0.144(1288/8939) | 1.00 | 0.06 | 1.00 | 0.18 |
|  | Bw4B- | 0.162(79/488) | 0.206(1000/4849) | 0.71 | 0.13 | **7.37x10^-3^** |  | 0.113(25/221) | 0.146(865/5905) | 0.73 | 0.22 | 0.14 |  |
| 2DS2+ | Bw4T80+ | 0.449(3232/7191) | 0.467(2467/5279) | 0.93 | 0.04 | **0.04** | **0.03** | 0.432(1358/3147) | 0.438(2892/6602) | 0.97 | 0.04 | 0.52 | 0.33 |
|  | Bw4T80- | 0.488(447/916) | 0.46(3187/6935) | 1.07 | 0.07 | 0.37 |  | 0.471(165/350) | 0.449(3704/8242) | 1.08 | 0.11 | 0.49 |  |
| 2DL3++ | Bw4T80+ | 0.551(3960/7191) | 0.533(2813/5279) | 1.08 | 0.04 | **0.05** | **0.03** | 0.569(1790/3147) | 0.562(3713/6602) | 1.03 | 0.04 | 0.54 | 0.34 |
|  | Bw4T80- | 0.512(469/916) | 0.54(3748/6935) | 0.94 | 0.07 | 0.37 |  | 0.529(185/350) | 0.55(4536/8242) | 0.93 | 0.11 | 0.49 |  |
| 2DL2+ | Bw4T80+ | 0.45(3234/7191) | 0.467(2467/5279) | 0.93 | 0.04 | **0.05** | **0.03** | 0.431(1356/3147) | 0.438(2891/6602) | 0.97 | 0.04 | 0.50 | 0.31 |
|  | Bw4T80- | 0.488(447/916) | 0.46(3187/6935) | 1.07 | 0.07 | 0.37 |  | 0.471(165/350) | 0.448(3694/8242) | 1.08 | 0.11 | 0.50 |  |
