# Supplementary material for: Epistatic interactions between killer immunoglobulin-like receptors and human leukocyte antigen ligands are associated with ankylosing spondylitis
Source: PLoS Genet. 2020 Aug 17;16(8):e1008906. doi: 10.1371/journal.pgen.1008906 (PMC7451988; doi:10.1371/journal.pgen.1008906)
Supplement: S8 Table — Interactions in blue are those between receptors and HLA ligands of a subclass known to biologically interact. Alternate shading indicates groups of KIRs (mostly in strong LD) that demonstrate statistical interactions with the same HLA allele. P = P-value denoting significance of the KIR association with AS when assessed in the specified HLA allele group, Int.P = P-value for the KIR-HLA allele interaction term, * = P-values that remain significant (P<0.05) after multiple testing correction, + = dominant inheritance, ++ = recessive inheritance (homozygosity), AS = ankylosing spondylitis, CTRL = control, OR = odds ratio, SE = standard error, NS = not significant, KIR2DS4D = KIR2DS4 deletion allele, KIR2DS4W = KIR2DS4 wild-type. Proportions are the proportion of individuals with the specified KIR genotype in cohorts split by HLA allele carriage. (DOCX) [file pgen.1008906.s008.docx]

|  |  | **TEST COHORT** | | | | | | **REPLICATION COHORT** | | | | | |
| --- | --- | --- | --- | --- | --- | --- | --- | --- | --- | --- | --- | --- | --- |
| **KIR** | **HLA** | **AS Prop. (Count)** | **CO Prop. (Count)** | **OR** | **SE** | **P** | **Int.Pval** | **AS Prop. (Count)** | **CO Prop. (Count)** | **OR** | **SE** | **P** | **Int.Pval** |
| *2DS2++* | *HLA-C*12+ (C1)* | 0.118(52/441) | 0.061(61/1000) | 2.05 | 0.20 | **3.67 x10^-4^*** | **5.12x10^-4^** | 0.076(11/144) | 0.063(65/1029) | 1.22 | 0.34 | 0.56 | 0.58 |
|  | *HLA-C*12- (C1)* | 0.07(533/7666) | 0.07(780/11214) | 0.99 | 0.06 | 0.90 |  | 0.066(221/3353) | 0.066(916/13815) | 0.99 | 0.08 | 0.89 |  |
| *2DL2++* | *HLA-C*12+ (C1)* | 0.118(52/441) | 0.061(61/1000) | 2.05 | 0.20 | **3.67 x10^-4^*** | **5.50x10^-4^** | 0.076(11/144) | 0.063(65/1029) | 1.22 | 0.34 | 0.56 | 0.63 |
|  | *HLA-C*12- (C1)* | 0.07(535/7666) | 0.07(780/11214) | 1.00 | 0.06 | 0.96 |  | 0.067(225/3353) | 0.066(910/13815) | 1.02 | 0.08 | 0.85 |  |
| *2DL3+* | *HLA-C*12+ (C1)* | 0.882(389/441) | 0.939(939/1000) | 0.49 | 0.20 | **3.67 x10^-4^*** | **5.52 x10^-4^** | 0.924(133/144) | 0.937(964/1029) | 0.82 | 0.34 | 0.56 | 0.62 |
|  | *HLA-C*12- (C1)* | 0.93(7131/7666) | 0.93(10434/11214) | 1.00 | 0.06 | 0.96 |  | 0.933(3129/3353) | 0.934(12904/13815) | 0.99 | 0.08 | 0.91 |  |
| *2DP1+* | *HLA-C*12+ (C1)* | 0.966(426/441) | 0.984(984/1000) | 0.46 | 0.37 | **0.03** | **0.02** | 0.993(143/144) | 0.983(1012/1029) | 2.28 | 1.04 | 0.43 | 0.25 |
|  | *HLA-C*12- (C1)* | 0.979(7505/7666) | 0.977(10951/11214) | 1.11 | 0.10 | 0.31 |  | 0.981(3289/3353) | 0.986(13616/13815) | 0.76 | 0.15 | 0.06 |  |
| *2DS4D+* | *HLA-C*12+ (C1)* | 0.864(381/441) | 0.812(812/1000) | 1.47 | 0.16 | **0.02** | **0.02** | 0.833(120/144) | 0.835(859/1029) | 1.00 | 0.24 | 0.99 | 0.71 |
|  | *HLA-C*12- (C1)* | 0.835(6402/7666) | 0.833(9338/11214) | 1.02 | 0.04 | 0.64 |  | 0.828(2777/3353) | 0.818(11301/13815) | 1.07 | 0.05 | 0.17 |  |
| *2DL1+* | *HLA-C*12+ (C1)* | 0.966(426/441) | 0.984(984/1000) | 0.46 | 0.37 | **0.03** | **0.03** | 0.993(143/144) | 0.984(1013/1029) | 2.16 | 1.04 | 0.46 | 0.33 |
|  | *HLA-C*12- (C1)* | 0.978(7496/7666) | 0.977(10953/11214) | 1.04 | 0.10 | 0.68 |  | 0.982(3293/3353) | 0.985(13603/13815) | 0.86 | 0.15 | 0.32 |  |
| *2DS1++* | *HLA-A*32+ (Bw4A)* | 0.014(11/786) | 0.039(34/866) | 0.32 | 0.36 | **1.44 x10^-3^*** | **1.87 x10^-3^** | 0.008(3/363) | 0.035(36/1025) | 0.23 | 0.61 | **0.02** | **0.01** |
|  | *HLA-A*32- (Bw4A)* | 0.038(279/7321) | 0.037(420/11348) | 1.02 | 0.08 | 0.76 |  | 0.037(115/3134) | 0.035(487/13819) | 1.04 | 0.11 | 0.69 |  |
| *3DL1+* | *HLA-A*32+ (Bw4A)* | 0.986(775/786) | 0.961(832/866) | 3.10 | 0.36 | **1.44 x10^-3^*** | **2.56 x10^-3^** | 0.992(360/363 | 0.965(989/1025) | 4.28 | 0.61 | **0.02** | **0.01** |
|  | *HLA-A*32- (Bw4A)* | 0.963(7052/7321) | 0.963(10930/11348) | 1.01 | 0.08 | 0.91 |  | 0.964(3020/3134) | 0.965(13332/13819) | 0.97 | 0.11 | 0.76 |  |
| *2DS4T+* | *HLA-A*32+ (Bw4A)* | 0.986(775/786) | 0.961(832/866) | 3.10 | 0.36 | **1.44 x10^-3^*** | **2.63 x10^-3^** | 0.992(360/363) | 0.965(989/1025) | 4.28 | 0.61 | **0.02** | **0.01** |
|  | *HLA-A*32- (Bw4A)* | 0.963(7052/7321) | 0.963(10929/11348) | 1.01 | 0.08 | 0.89 |  | 0.963(3019/3134) | 0.965(13332/13819) | 0.96 | 0.11 | 0.69 |  |
| *3DS1++* | *HLA-A*32+ (Bw4A)* | 0.02(16/786) | 0.047(41/866) | 0.40 | 0.30 | **2.22 x10^-3^*** | **5.19 x10^-3^** | 0.011(4/363) | 0.042(43/1025) | 0.26 | 0.53 | **0.01** | **9.38 x10^-3^** |
|  | *HLA-A*32- (Bw4A)* | 0.042(304/7321) | 0.043(487/11348) | 0.96 | 0.08 | 0.61 |  | 0.041(128/3134) | 0.04(551/13819) | 1.02 | 0.10 | 0.81 |  |
| *2DS5++* | *HLA-A*32+ (Bw4A)* | 0.009(7/786) | 0.024(21/866) | 0.35 | 0.44 | **0.02** | **0.01** | 0.008(3/363) | 0.027(28/1025) | 0.31 | 0.61 | **0.05** | 0.06 |
|  | *HLA-A*32- (Bw4A)* | 0.023(166/7321) | 0.021(236/11348) | 1.09 | 0.10 | 0.42 |  | 0.021(66/3134) | 0.022(302/13819) | 0.96 | 0.14 | 0.78 |  |
| *2DS3+* | *HLA-C*02+ (C2)* | 0.206(745/3612) | 0.166(162/975) | 1.30 | 0.10 | **5.46 x10^-3^** | **1.93 x10^-3^** | 0.138(205/1485) | 0.138(146/1060) | 0.99 | 0.12 | 0.90 | 0.98 |
|  | *HLA-C*02- (C2)* | 0.199(893/4495) | 0.21(2356/11239) | 0.93 | 0.04 | 0.09 |  | 0.145(291/2012) | 0.146(2007/13784) | 0.99 | 0.07 | 0.92 |  |
| *2DP1++* | *HLA-B*07+ (Bw6)* | 0.76(797/1049) | 0.709(2213/3120) | 1.30 | 0.08 | **1.39 x10^-3^*** | **2.13 x10^-3^** | 0.79(373/472) | 0.775(3189/4116) | 1.10 | 0.12 | 0.42 | 0.43 |
|  | *HLA-B*07- (Bw6)* | 0.72(5080/7058) | 0.723(6578/9094) | 0.98 | 0.04 | 0.57 |  | 0.771(2332/3025) | 0.772(8281/10728) | 0.99 | 0.05 | 0.92 |  |
| *2DL1++* | *HLA-B*07+ (Bw6)* | 0.749(786/1049) | 0.707(2206/3120) | 1.25 | 0.08 | **7.28 x10^-3^** | **8.67 x10^-3^** | 0.794(375/472) | 0.769(3165/4116) | 1.17 | 0.12 | 0.20 | 0.37 |
|  | *HLA-B*07- (Bw6)* | 0.713(5030/7058) | 0.717(6517/9094) | 0.98 | 0.04 | 0.52 |  | 0.773(2338/3025) | 0.766(8218/10728) | 1.04 | 0.05 | 0.43 |  |
| *2DS4T++* | *HLA-C*05+ (C2)* | 0.637(627/985) | 0.672(1696/2525) | 0.86 | 0.08 | **0.05** | **2.41 x10^-3^** | 0.637(319/501) | 0.665(2114/3181) | 0.88 | 0.10 | 0.22 | 0.09 |
|  | *HLA-C*05- (C2)* | 0.679(4833/7122) | 0.657(6365/9689) | 1.11 | 0.03 | **1.83 x10^-3^*** |  | 0.685(2051/2996) | 0.67(7813/11663) | 1.07 | 0.04 | 0.13 |  |
| *3DL1++* | *HLA-C*05+ (C2)* | 0.637(627/985) | 0.672(1696/2525) | 0.86 | 0.08 | **0.05** | **2.47 x10^-3^** | 0.637(319/501) | 0.665(2114/3181) | 0.88 | 0.10 | 0.22 | 0.10 |
|  | *HLA-C*05- (C2)* | 0.679(4835/7122) | 0.657(6369/9689) | 1.11 | 0.03 | **1.94 x10^-3^*** |  | 0.685(2051/296) | 0.671(7822/11663) | 1.07 | 0.04 | 0.15 |  |
| *2DS1+* | *HLA-C*05+ (C2)* | 0.363(358/985) | 0.33(833/2525) | 1.16 | 0.08 | 0.06 | **3.16 x10^-3^** | 0.363(182/501) | 0.335(1067/3181) | 1.13 | 0.10 | 0.22 | 0.09 |
|  | *HLA-C*05- (C2)* | 0.321(2287/7122) | 0.343(3322/9689) | 0.90 | 0.03 | **1.76 x10^-3^*** |  | 0.315(945/2996) | 0.33(3846/11663) | 0.94 | 0.04 | 0.14 |  |
| *3DS1+* | *HLA-C*05+ (C2)* | 0.36(355/985) | 0.328(828/2525) | 1.15 | 0.08 | 0.07 | **4.78 x10^-3^** | 0.355(178/501) | 0.333(1058/3181) | 1.11 | 0.10 | 0.31 | 0.16 |
|  | *HLA-C*05- (C2)* | 0.32(2281/7122) | 0.341(3300/9689) | 0.91 | 0.03 | **3.37 x10^-3^*** |  | 0.315(943/2996) | 0.327(3817/11663) | 0.94 | 0.04 | 0.19 |  |
| *2DS4D+* | *HLA-C*05+ (C2)* | 0.813(801/985) | 0.843(2129/2525) | 0.81 | 0.10 | **0.03** | **4.92 x10^-3^** | 0.828(415/501) | 0.817(2598/3181) | 1.09 | 0.13 | 0.52 | 0.88 |
|  | *HLA-C*05- (C2)* | 0.84(5982/7122) | 0.828(8021/9689) | 1.09 | 0.04 | **0.03** |  | 0.828(2482/2996) | 0.82(9562/11663) | 1.06 | 0.05 | 0.28 |  |
| *2DL5+* | *HLA-C*05+ (C2)* | 0.467(460/985) | 0.439(1109/2525) | 1.11 | 0.08 | 0.16 | **0.01** | 0.413(207/501) | 0.4(1272/3181) | 1.06 | 0.10 | 0.58 | 0.20 |
|  | *HLA-C*05- (C2)* | 0.433(3082/7122) | 0.452(4384/9689) | 0.91 | 0.03 | **4.31 x10^-3^** |  | 0.373(1119/2996) | 0.394(4590/11663) | 0.92 | 0.04 | 0.05 |  |
| *2DL3+* | *HLA-A*31+* | 0.953(634/665) | 0.918(583/635) | 1.90 | 0.24 | **6.57 x10^-3^** | **3.73 x10^-3^** | 0.978(272/278) | 0.921(737/800) | 3.95 | 0.43 | **1.57x10^-3^*** | **9.33 x10^-4^** |
|  | *HLA-A*31-* | 0.925(6886/7442) | 0.932(10790/11579) | 0.91 | 0.06 | 0.11 |  | 0.929(2990/3219) | 0.935(13131/14044) | 0.91 | 0.08 | 0.24 |  |
| *2DL2++* | *HLA-A*31+* | 0.047(31/665) | 0.082(52/635) | 0.53 | 0.24 | **6.57 x10^-3^** | **3.74 x10^-3^** | 0.022(6/278) | 0.078(62/800) | 0.26 | 0.44 | **1.77x10^-3^*** | **1.04 x10^-3^** |
|  | *HLA-A*31-* | 0.075(556/7442) | 0.068(789/11579) | 1.10 | 0.06 | 0.11 |  | 0.071(230/3219) | 0.065(913/14044) | 1.10 | 0.08 | 0.21 |  |
| *2DS2++* | *HLA-A*31+* | 0.047(31/665) | 0.082(52/635) | 0.53 | 0.24 | **6.57 x10^-3^** | **3.94 x10^-3^** | 0.022(6/278) | 0.08(64/800) | 0.25 | 0.43 | **1.35x10^-3^*** | **9.47 x10^-4^** |
|  | *HLA-A*31-* | 0.074(554/7442) | 0.068(789/11579) | 1.09 | 0.06 | 0.12 |  | 0.07(226/3219) | 0.065(917/14044) | 1.07 | 0.08 | 0.35 |  |
| *2DS3++* | *HLA-A*31+* | 0.033(22/665) | 0.061(39/635) | 0.51 | 0.28 | **0.02** | **0.02** | 0.025(7/278) | 0.044(35/800) | 0.55 | 0.42 | 0.16 | **0.20** |
|  | *HLA-A*31-* | 0.046(342/7442) | 0.046(535/11579) | 1.00 | 0.07 | 0.97 |  | 0.042(135/3219) | 0.042(583/14044) | 1.01 | 0.10 | 0.89 |  |
| *2DL5+* | *HLA-B*49+ (Bw4BI80)* | 0.57(65/114) | 0.438(140/320) | 1.67 | 0.23 | **0.02** | **6.34 x10^-3^** | 0.5(16/32) | 0.375(116/309) | 1.84 | 0.39 | 0.12 | 0.13 |
|  | *HLA-B*49- (Bw4BI80)* | 0.435(3477/7993) | 0.45(5353/11894) | 0.93 | 0.03 | **0.02** |  | 0.378(1310/3465) | 0.395(5746/14535) | 0.93 | 0.04 | 0.06 |  |
| *2DS5+* | *HLA-B*49+ (Bw4BI80)* | 0.333(38/114) | 0.241(77/320) | 1.66 | 0.25 | **0.04** | **0.03** | 0.438(14/32) | 0.23(71/309) | 2.85 | 0.40 | **8.34x10^-3^** | **7.63x10^-3^** |
|  | *HLA-B*49- (Bw4BI80)* | 0.254(2033/7993) | 0.263(3132/11894) | 0.95 | 0.03 | 0.13 |  | 0.254(880/3465) | 0.268(3890/14535) | 0.93 | 0.04 | 0.10 |  |
| *2DL5+* | *HLA-B*27+ (Bw4BT80)* | 0.437(3041/6952) | 0.4(427/1067) | 1.16 | 0.07 | **0.03** | **7.35 x10^-3^** | 0.381(1134/2974) | 0.398(487/1225) | 0.93 | 0.07 | 0.29 | 0.71 |
|  | *HLA-B*27- (Bw4BT80)* | 0.434(501/1155) | 0.454(5066/11147) | 0.90 | 0.06 | 0.08 |  | 0.367(192/523) | 0.395(5375/13619) | 0.89 | 0.09 | 0.20 |  |
| *2DS4T++* | *HLA-B*27+ (Bw4BT80)* | 0.673(4678/6952) | 0.707(754/1067) | 0.85 | 0.07 | **0.03** | **8.78 x10^-3^** | 0.674(2004/2974) | 0.669(820/1225) | 1.03 | 0.07 | 0.70 | 0.33 |
|  | *HLA-B*27- (Bw4BT80)* | 0.677(782/1155) | 0.656(7307/11147) | 1.11 | 0.07 | 0.13 |  | 0.7(366/523) | 0.669(9107/13619) | 1.16 | 0.10 | 0.13 |  |
| *2DS1+* | *HLA-B*27+ (Bw4BT80)* | 0.327(2272/6952) | 0.293(313/1067) | 1.17 | 0.07 | **0.03** | **8.90 x10^-3^** | 0.326(970/2974) | 0.33(404/1225) | 0.98 | 0.07 | 0.73 | 0.32 |
|  | *HLA-B*27- (Bw4BT80)* | 0.323(373/1155) | 0.345(3842/11147) | 0.90 | 0.07 | 0.13 |  | 0.3(157/523) | 0.331(4509/13619) | 0.87 | 0.10 | 0.14 |  |
| *3DL1++* | *HLA-B*27+ (Bw4BT80)* | 0.673(4680/6952) | 0.707(754/1067) | 0.85 | 0.07 | **0.03** | **0.01** | 0.674(2004/2974) | 0.670(821/1225) | 1.02 | 0.07 | 0.73 | 0.33 |
|  | *HLA-B*27- (Bw4BT80)* | 0.677(782/1155) | 0.656(7311/11147) | 1.10 | 0.07 | 0.14 |  | 0.7(366/523) | 0.669(9115/13619) | 1.15 | 0.10 | 0.14 |  |
| *3DS1+* | *HLA-B*27+ (Bw4BT80)* | 0.325(2260/6952) | 0.292(312/1067) | 1.17 | 0.07 | **0.03** | **0.02** | 0.324(963/2974) | 0.328(402/1225) | 0.97 | 0.07 | 0.70 | 0.43 |
|  | *HLA-B*27- (Bw4BT80)* | 0.326(376/1155) | 0.342(3816/11147) | 0.93 | 0.07 | 0.25 |  | 0.302(158/523) | 0.328(4473/13619) | 0.88 | 0.10 | 0.20 |  |
| *2DS5+* | *HLA-B*27+ (Bw4BT80)* | 0.256(1780/6952) | 0.227(242/1067) | 1.18 | 0.08 | **0.03** | **0.02** | 0.258(766/2974) | 0.268(328/1225) | 0.95 | 0.08 | 0.49 | 0.62 |
|  | *HLA-B*27- (Bw4BT80)* | 0.252(291/1155) | 0.266(2967/11147) | 0.93 | 0.07 | 0.28 |  | 0.245(128/523) | 0.267(3633/13619) | 0.89 | 0.10 | 0.25 |  |
| *2DS5++* | *HLA-B*27+ (Bw4BT80)* | 0.022(153/6952) | 0.012(13/1067) | 1.82 | 0.29 | **0.04** | **0.02** | 0.02(58/2974) | 0.024(30/1225) | 0.79 | 0.23 | 0.30 | 0.64 |
|  | *HLA-B*27- (Bw4BT80)* | 0.017(20/1155) | 0.022(244/11147) | 0.77 | 0.24 | 0.27 |  | 0.021(11/523) | 0.022(300/13619) | 0.94 | 0.31 | 0.85 |  |
| *2DS2++* | *HLA-A*30+* | 0.102(27/265) | 0.05(27/535) | 2.30 | 0.29 | **4.09 x10^-3^*** | **0.01** | 0.105(9/86) | 0.07(41/586) | 1.48 | 0.40 | 0.32 | 0.26 |
|  | *HLA-A*30-* | 0.071(558/7842) | 0.07(814/11679) | 1.02 | 0.06 | 0.76 |  | 0.065(223/3411) | 0.066(940/14258) | 0.99 | 0.08 | 0.85 |  |
| *2DL2++* | *HLA-A*30+* | 0.102(27/265) | 0.05(27/535) | 2.30 | 0.29 | **4.09 x10^-3^*** | **0.01** | 0.105(9/86) | 0.07(41/586) | 1.48 | 0.40 | 0.32 | 0.29 |
|  | *HLA-A*30-* | 0.071(560/7842) | 0.07(814/11679) | 1.02 | 0.06 | 0.71 |  | 0.067(227/3411) | 0.066(934/14258) | 1.01 | 0.08 | 0.88 |  |
| *2DL3+* | *HLA-A*30+* | 0.898(238/265) | 0.95(508/535) | 0.43 | 0.29 | **4.09 x10^-3^*** | **0.01** | 0.895(77/86) | 0.93(545/586) | 0.67 | 0.40 | 0.32 | 0.29 |
|  | *HLA-A*30-* | 0.929(7282/7842) | 0.93(10865/11679) | 0.98 | 0.06 | 0.71 |  | 0.934(3185/3411) | 0.934(13323/14258) | 0.99 | 0.08 | 0.95 |  |
| *2DL5+* | *HLA-A*30+* | 0.502(133/265) | 0.426(228/535) | 1.31 | 0.15 | 0.08 | **0.02** | 0.407(35/86) | 0.415(243/586) | 0.94 | 0.24 | 0.80 | 0.86 |
|  | *HLA-A*30-* | 0.435(3409/7842) | 0.451(5265/11679) | 0.93 | 0.03 | **0.01** |  | 0.378(1291/3411) | 0.394(5619/14258) | 0.94 | 0.04 | 0.09 |  |
| *2DS5+* | *HLA-B*40+ (Bw6)* | 0.219(205/935) | 0.267(424/1589) | 0.75 | 0.10 | **3.93 x10^-3^*** | **0.01** | 0.202(83/411) | 0.265(508/1914) | 0.70 | 0.13 | **6.76x10^-3^** | **0.02** |
|  | *HLA-B*40- (Bw6)* | 0.26(1866/7172) | 0.262(2785/10625) | 0.99 | 0.04 | 0.73 |  | 0.263(811/3086) | 0.267(3453/12930) | 0.98 | 0.05 | 0.64 |  |
| *2DL5+* | *HLA-B*40+ (Bw6)* | 0.399(373/935) | 0.452(719/1589) | 0.78 | 0.09 | **3.63 x10^-3^*** | **0.03** | 0.328(135/411) | 0.393(753/1914) | 0.75 | 0.12 | **0.01** | **0.05** |
|  | *HLA-B*40- (Bw6)* | 0.442(3169/7172) | 0.449(4774/10625) | 0.96 | 0.03 | 0.22 |  | 0.386(1191/3086) | 0.395(5109/12930) | 0.96 | 0.04 | 0.35 |  |
| *2DS1+* | *HLA-C*07+ (C1)* | 0.32(888/2774) | 0.351(2393/6810) | 0.86 | 0.05 | **2.37 x10^-3^*** | **0.01** | 0.308(404/1312) | 0.33(2825/8553) | 0.91 | 0.06 | 0.13 | 0.25 |
|  | *HLA-C*07- (C1)* | 0.329(1757/5333) | 0.326(1762/5404) | 1.01 | 0.04 | 0.81 |  | 0.331(723/2185) | 0.332(2088/6291) | 1.00 | 0.05 | 0.94 |  |
| *2DS4T++* | *HLA-C*07+ (C1)* | 0.679(1884/2774) | 0.648(4416/6810) | 1.15 | 0.05 | **2.83 x10^-3^*** | **0.02** | 0.692(908/1312) | 0.669(5726/8553) | 1.10 | 0.06 | 0.12 | 0.26 |
|  | *HLA-C*07- (C1)* | 0.671(3576/5333) | 0.675(3645/5404) | 0.99 | 0.04 | 0.77 |  | 0.669(1462/2185) | 0.668(4201/6291) | 1.01 | 0.05 | 0.92 |  |
| *3DL1++* | *HLA-C*07+ (C1)* | 0.679(1884/2774) | 0.649(4418/6810) | 1.15 | 0.05 | **3.09 x10^-3^*** | **0.02** | 0.692(980/1312) | 0.67(5732/8553) | 1.10 | 0.06 | 0.14 | 0.26 |
|  | *HLA-C*07- (C1)* | 0.671(3578/5333) | 0.675(3647/5404) | 0.99 | 0.04 | 0.77 |  | 0.669(1462/2185) | 0.668(4204/6219) | 1.00 | 0.05 | 0.95 |  |
| *3DS1+* | *HLA-C*07+ (C1)* | 0.32(887/2774) | 0.349(2379/6810) | 0.87 | 0.05 | **3.90 x10^-3^*** | **0.02** | 0.307(403/1312) | 0.328(2802/8553) | 0.91 | 0.06 | 0.16 | 0.29 |
|  | *HLA-C*07- (C1)* | 0.328(1749/5333) | 0.324(1749/5404) | 1.01 | 0.04 | 0.73 |  | 0.329(718/2185) | 0.33(2073/6291) | 1.00 | 0.05 | 0.95 |  |
| *2DL3++* | *HLA-C*07+ (C1)* | 0.569(1578/2774) | 0.538(3661/6810) | 1.14 | 0.05 | **3.36 x10^-3^*** | **0.02** | 0.585(767/1312) | 0.554(4735/8553) | 1.14 | 0.06 | **0.03** | **0.05** |
|  | *HLA-C*07- (C1)* | 0.535(2851/5333) | 0.537(2900/5404) | 0.99 | 0.04 | 0.79 |  | 0.553(1208/2185) | 0.559(3514/6291) | 0.98 | 0.05 | 0.65 |  |
| *2DL2+* | *HLA-C*07+ (C1)* | 0.432(1197/2774) | 0.462(3149/6810) | 0.88 | 0.05 | **3.72 x10^-3^*** | **0.02** | 0.416(546/1312) | 0.445(3809/8553) | 0.89 | 0.06 | 0.04 | 0.07 |
|  | *HLA-C*07- (C1)* | 0.466(2484/5333) | 0.464(2505/5404) | 1.01 | 0.04 | 0.778 |  | 0.446(975/2185) | 0.441(2776/6291) | 1.02 | 0.05 | 0.69 |  |
| *2DS2+* | *HLA-C*07+ (C1)* | 0.432(1197/2774) | 0.462(3149/6810) | 0.88 | 0.05 | **3.72 x10^-3^*** | **0.02** | 0.415(545/1312) | 0.447(3819/8553) | 0.88 | 0.06 | **0.03** | **0.05** |
|  | *HLA-C*07- (C1)* | 0.465(2482/5333) | 0.464(2505/5404) | 1.01 | 0.04 | 0.81 |  | 0.448(978/2185) | 0.441(2777/6291) | 1.02 | 0.05 | 0.63 |  |
| *2DL5+* | *HLA-C*07+ (C1)* | 0.428(1186/2774) | 0.459(3123/6810) | 0.87 | 0.05 | **3.26 x10^-3^*** | **0.02** | 0.364(478/1312) | 0.395(3378/8553) | 0.88 | 0.06 | **0.04** | 0.23 |
|  | *HLA-C*07- (C1)* | 0.442(2356/5333) | 0.439(2370/5404) | 1.00 | 0.04 | 0.90 |  | 0.388(848/2185) | 0.395(2484/6291) | 0.97 | 0.05 | 0.59 |  |
| *2DP1++* | *HLA-C*07+ (C1)* | 0.743(2060/2774) | 0.719(4897/6810) | 1.13 | 0.05 | **0.02** | **0.03** | 0.789(1035/1312) | 0.77(6584/8553) | 1.12 | 0.07 | 0.12 | **0.05** |
|  | *HLA-C*07- (C1)* | 0.716(3817/5333) | 0.721(3894/5404) | 0.97 | 0.04 | 0.52 |  | 0.764(1670/2185) | 0.777(4886/6291) | 0.93 | 0.06 | 0.25 |  |
| *2DS5+* | *HLA-C*07+ (C1)* | 0.256(711/2774) | 0.276(1880/6810) | 0.90 | 0.05 | **0.04** | **0.03** | 0.254(333/1312) | 0.267(2280/8553) | 0.94 | 0.07 | 0.36 | 0.91 |
|  | *HLA-C*07- (C1)* | 0.255(1360/5333) | 0.246(1329/5404) | 1.04 | 0.05 | 0.35 |  | 0.257(561/2185) | 0.267(1681/6291) | 0.95 | 0.06 | 0.36 |  |
| *2DL5+* | *HLA-A*03+* | 0.407(748/1837) | 0.45(1469/3261) | 0.83 | 0.06 | **1.64 x10^-3^*** | **0.02** | 0.374(313/837) | 0.405(1599/3947) | 0.88 | 0.08 | 0.10 | 0.32 |
|  | *HLA-A*03-* | 0.446(2794/6270) | 0.449(4024/8953) | 0.98 | 0.03 | 0.48 |  | 0.381(1013/2660) | 0.391(4263/10897) | 0.96 | 0.05 | 0.34 |  |
| *3DS1++* | *HLA-C*01+ (C1)* | 0.047(152/3258) | 0.034(28/814) | 1.37 | 0.21 | 0.14 | **0.02** | 0.047(66/1393) | 0.045(43/960) | 1.06 | 0.20 | 0.77 | 0.20 |
|  | *HLA-C*01- (C1)* | 0.035(168/4849) | 0.044(500/11400) | 0.78 | 0.09 | **5.39 x10^-3^** |  | 0.031(66/2104) | 0.04(551/13884) | 0.78 | 0.13 | 0.06 |  |
| *2DS4D+* | *HLA-C*01+ (C1)* | 0.826(2692/3258) | 0.848(690/814) | 0.85 | 0.11 | 0.15 | **0.02** | 0.819(1141/1393) | 0.827(794/960) | 0.95 | 0.11 | 0.67 | 0.21 |
|  | *HLA-C*01- (C1)* | 0.844(4091/4849) | 0.83(9460/11400) | 1.11 | 0.05 | **0.02** |  | 0.835(1756/2104) | 0.819(11366/13884) | 1.12 | 0.06 | 0.08 |  |
| *2DS4D++* | *HLA-C*01+ (C1)* | 0.362(1178/3258) | 0.382(311/814) | 0.92 | 0.08 | 0.32 | **0.04** | 0.341(475/1393) | 0.369(354/960) | 0.89 | 0.09 | 0.20 | **0.03** |
|  | *HLA-C*01- (C1)* | 0.374(1814/4849) | 0.352(4012/11400) | 1.10 | 0.04 | **5.99 x10^-3^** |  | 0.37(779/2104) | 0.346(4809/13884) | 1.11 | 0.05 | **0.03** |  |
| *2DS1++* | *HLA-C*01+ (C1)* | 0.042(137/3258) | 0.031(25/814) | 1.37 | 0.22 | 0.16 | **0.04** | 0.039(55/1393) | 0.038(36/960) | 1.05 | 0.22 | 0.84 | 0.42 |
|  | *HLA-C*01- (C1)* | 0.032(153/4849) | 0.038(429/11400) | 0.82 | 0.10 | **0.04** |  | 0.03(63/2104) | 0.035(487/13884) | 0.85 | 0.14 | 0.23 |  |
| *2DS4T+* | *HLA-C*01+ (C1)* | 0.96(3127/3258) | 0.969(789/814) | 0.77 | 0.22 | 0.23 | **0.05** | 0.961(1338/1393) | 0.962(924/960) | 0.96 | 0.22 | 0.84 | 0.42 |
|  | *HLA-C*01- (C1)* | 0.969(4700/4849) | 0.962(10972/11400) | 1.24 | 0.10 | **0.02** |  | 0.97(2041/2104) | 0.965(13397/13884) | 1.18 | 0.14 | 0.23 |  |
| *2DS4T++* | *HLA-C*01+ (C1)* | 0.676(2201/3258) | 0.699(569/814) | 0.90 | 0.09 | 0.22 | **0.05** | 0.665(926/1393) | 0.68(653/960) | 0.93 | 0.09 | 0.45 | 0.14 |
|  | *HLA-C*01- (C1)* | 0.672(3259/4849) | 0.657(7492/11400) | 1.08 | 0.04 | **0.04** |  | 0.686(1444/2104) | 0.668(9274/13884) | 1.09 | 0.05 | 0.09 |  |
| *2DL1+* | *HLA-A*24+ (Bw4A)* | 0.969(1264/1304) | 0.981(1900/1936) | 0.58 | 0.23 | **0.02** | **0.02** | 0.986(501/508) | 0.985(2135/2167) | 1.07 | 0.42 | 0.87 | 0.62 |
|  | *HLA-A*24- (Bw4A)* | 0.979(6658/6803) | 0.977(10037/10278) | 1.09 | 0.11 | 0.40 |  | 0.982(2935/2989) | 0.985(12481/12677) | 0.86 | 0.16 | 0.34 |  |
| *2DP1+* | *HLA-A*24+ (Bw4A)* | 0.971(1266/1304) | 0.981(1899/1936) | 0.63 | 0.24 | **0.05** | **0.02** | 0.984(500/508) | 0.985(2135/2167) | 0.94 | 0.40 | 0.87 | 0.63 |
|  | *HLA-A*24- (Bw4A)* | 0.98(6665/6803) | 0.976(10036/10278) | 1.15 | 0.11 | 0.18 |  | 0.981(2932/2989) | 0.985(12493/12677) | 0.76 | 0.15 | 0.08 |  |
| *3DS1++* | *HLA-A*24+ (Bw4A)* | 0.034(44/1304) | 0.052(100/1936) | 0.63 | 0.19 | **0.01** | **0.04** | 0.031(16/508) | 0.036(79/2167) | 0.85 | 0.28 | 0.55 | 0.70 |
|  | *HLA-A*24- (Bw4A)* | 0.041(276/6803) | 0.042(428/10278) | 0.97 | 0.08 | 0.69 |  | 0.039(116/2989) | 0.041(515/12677) | 0.95 | 0.11 | 0.66 |  |
| *3DL1+* | *HLA-A*24+ (Bw4A)* | 0.971(1266/1304) | 0.955(1849/1936) | 1.56 | 0.20 | **0.03** | **0.04** | 0.972(494/508) | 0.967(2096/2167) | 1.21 | 0.30 | 0.52 | 0.63 |
|  | *HLA-A*24- (Bw4A)* | 0.964(6561/6803) | 0.964(9913/10278) | 1.01 | 0.09 | 0.94 |  | 0.966(2886/2989) | 0.964(12225/12677) | 1.03 | 0.11 | 0.77 |  |
| *2DS4T+* | *HLA-A*24+ (Bw4A)* | 0.971(1266/1304) | 0.955(1849/1936) | 1.56 | 0.20 | **0.03** | **0.04** | 0.97(493/508) | 0.967(2096/2167) | 1.13 | 0.29 | 0.68 | 0.79 |
|  | *HLA-A*24- (Bw4A)* | 0.964(6561/6803) | 0.964(9912/10278) | 1.01 | 0.09 | 0.91 |  | 0.966(2886/2989) | 0.964(12225/12677) | 1.03 | 0.11 | 0.77 |  |
| *2DS1++* | *HLA-A*24+ (Bw4A)* | 0.031(40/1304) | 0.045(87/1936) | 0.68 | 0.20 | **0.05** | **0.05** | 0.03(15/508) | 0.033(71/2167) | 0.89 | 0.29 | 0.68 | 0.79 |
|  | *HLA-A*24- (Bw4A)* | 0.037(250/6803) | 0.036(367/10278) | 1.02 | 0.08 | 0.80 |  | 0.034(103/2989) | 0.036(452/12677) | 0.97 | 0.11 | 0.77 |  |
| *2DS4W+* | *HLA-A*11+* | 0.342(360/1052) | 0.397(577/1453) | 0.79 | 0.09 | **6.81 x10^-3^** | **0.02** | 0.367(169/460) | 0.421(757/1797) | 0.79 | 0.11 | **0.03** | **0.05** |
|  | *HLA-A*11-* | 0.39(2752/7055) | 0.394(4235/10761) | 0.99 | 0.03 | 0.69 |  | 0.409(1241/3037) | 0.409(5341/13047) | 1.00 | 0.04 | 0.93 |  |
| *2DS3+* | *HLA-B*37+ (Bw4BT80)* | 0.126(14/111) | 0.239(82/343) | 0.43 | 0.32 | **8.70 x10^-3^** | **0.02** | 0.152(7/46) | 0.127(54/425) | 1.39 | 0.46 | 0.47 | 0.58 |
|  | *HLA-B*37- (Bw4BT80)* | 0.203(1624/7996) | 0.205(2436/11871) | 0.98 | 0.04 | 0.57 |  | 0.142(489/3451) | 0.146(2099/14419) | 0.97 | 0.05 | 0.57 |  |
| *2DS5+* | *HLA-B*51+ (Bw4BI80)* | 0.281(139/495) | 0.235(238/1013) | 1.28 | 0.13 | **0.05** | **0.02** | 0.315(63/200) | 0.281(318/1132) | 1.18 | 0.17 | 0.33 | 0.19 |
|  | *HLA-B*51- (Bw4BI80)* | 0.254(1932/7612) | 0.265(2971/11201) | 0.94 | 0.03 | 0.06 |  | 0.252(831/3297) | 0.266(3643/13712) | 0.93 | 0.05 | 0.13 |  |
| *3DS1+* | *HLA-B*51+ (Bw4BI80)* | 0.349(173/495) | 0.307(311/1013) | 1.22 | 0.12 | 0.08 | **0.02** | 0.375(75/200) | 0.335(379/1132) | 1.19 | 0.16 | 0.27 | 0.19 |
|  | *HLA-B*51- (Bw4BI80)* | 0.324(2463/7612) | 0.341(3817/11201) | 0.92 | 0.03 | **8.36 x10^-3^** |  | 0.317(1046/3297) | 0.328(4496/13712) | 0.95 | 0.04 | 0.25 |  |
| *2DS1+* | *HLA-B*51+ (Bw4BI80)* | 0.349(173/495) | 0.309(313/1013) | 1.21 | 0.12 | 0.10 | **0.02** | 0.375(75/200) | 0.337(381/1132) | 1.19 | 0.16 | 0.29 | 0.20 |
|  | *HLA-B*51- (Bw4BI80)* | 0.325(2472/7612) | 0.343(3842/11201) | 0.92 | 0.03 | **5.18 x10^-3^** |  | 0.319(1052/3297) | 0.331(4532/13712) | 0.95 | 0.04 | 0.21 |  |
| *2DS4T++* | *HLA-B*51+ (Bw4BI80)* | 0.651(322/495) | 0.691(700/1013) | 0.82 | 0.12 | 0.10 | **0.02** | 0.625(125/200) | 0.663(751/1132) | 0.84 | 0.16 | 0.29 | 0.19 |
|  | *HLA-B*51- (Bw4BI80)* | 0.675(5138/7612) | 0.657(7361/11201) | 1.09 | 0.03 | **6.31 x10^-3^** |  | 0.681(2245/3297) | 0.669(9176/13712) | 1.05 | 0.04 | 0.20 |  |
| *3DL1++* | *HLA-B*51+ (Bw4BI80)* | 0.651(322/495) | 0.691(700/1013) | 0.82 | 0.12 | 0.10 | **0.03** | 0.625(125/200) | 0.663(751/1132) | 0.84 | 0.16 | 0.29 | 0.20 |
|  | *HLA-B*51- (Bw4BI80)* | 0.675(5140/7612) | 0.658(7365/11201) | 1.09 | 0.03 | **6.59 x10^-3^** |  | 0.681(2245/3297) | 0.670(9185/13712) | 1.05 | 0.04 | 0.23 |  |
| *2DS4D+* | *HLA-B*44+ (Bw4BT80)* | 0.819(1147/1401) | 0.837(3113/3720) | 0.88 | 0.08 | 0.13 | **0.02** | 0.814(583/716) | 0.82(3934/4797) | 0.96 | 0.10 | 0.69 | 0.26 |
|  | *HLA-B*44- (Bw4BT80)* | 0.84(5636/6706) | 0.828(7037/8494) | 1.09 | 0.04 | **0.04** |  | 0.832(2314/2781) | 0.819(8226/10047) | 1.10 | 0.06 | 0.12 |  |
| *2DL3++* | *HLA-B*52+ (Bw4BI80)* | 0.382(29/76) | 0.531(77/145) | 0.51 | 0.30 | **0.03** | **0.02** | 0.458(11/24) | 0.464(84/181) | 1.14 | 0.46 | 0.78 | 0.90 |
|  | *HLA-B*52- (Bw4BI80)* | 0.548(4400/8031) | 0.537(6484/12069) | 1.05 | 0.03 | 0.11 |  | 0.566(1964/3473) | 0.557(8165/14663) | 1.04 | 0.04 | 0.33 |  |
| *2DS4D+* | *HLA-B*52+ (Bw4BI80)* | 0.895(68/76) | 0.766(111/145) | 2.69 | 0.44 | **0.02** | **0.03** | 0.875(21/24) | 0.845(153/181) | 1.11 | 0.70 | 0.88 | 0.79 |
|  | *HLA-B*52- (Bw4BI80)* | 0.836(6715/8031) | 0.832(10039/12069) | 1.03 | 0.04 | 0.39 |  | 0.828(2876/3473) | 0.819(12007/14663) | 1.06 | 0.05 | 0.21 |  |
| *2DS2+* | *HLA-B*52+ (Bw4BI80)* | 0.618(47/76) | 0.476(69/145) | 1.89 | 0.30 | **0.03** | **0.03** | 0.542(13/24) | 0.53(96/181) | 0.88 | 0.46 | 0.79 | 0.86 |
|  | *HLA-B*52- (Bw4BI80)* | 0.452(3632/8031) | 0.463(5585/12069) | 0.96 | 0.03 | 0.12 |  | 0.435(1510/3473) | 0.443(6500/14663) | 0.96 | 0.04 | 0.33 |  |
| *2DL2+* | *HLA-B*52+ (Bw4BI80)* | 0.618(47/76) | 0.476(69/145) | 1.89 | 0.30 | **0.03** | **0.03** | 0.542(13/24) | 0.53(96/181) | 0.88 | 0.46 | 0.79 | 0.86 |
|  | *HLA-B*52- (Bw4BI80)* | 0.452(3634/8031) | 0.463(5585/12069) | 0.96 | 0.03 | 0.13 |  | 0.434(1508/3473) | 0.443(6489/14663) | 0.96 | 0.04 | 0.35 |  |
| *2DL1+* | *HLA-A*23+ (Bw4A)* | 0.995(183/184) | 0.953(368/386) | 10.05 | 1.04 | **0.03** | **0.03** | 0.969(62/64) | 0.984(494/502) | 0.46 | 0.83 | 0.36 | 0.52 |
|  | *HLA-A*23- (Bw4A)* | 0.977(7739/7923) | 0.978(11569/11828) | 0.93 | 0.10 | 0.47 |  | 0.983(3374/3433) | 0.985(14122/14342) | 0.90 | 0.15 | 0.47 |  |
| *2DP1+* | *HLA-A*23+ (Bw4A)* | 0.995(183/184) | 0.959(370/386) | 8.49 | 1.04 | **0.04** | **0.04** | 0.953(61/64) | 0.984(494/502) | 0.31 | 0.73 | 0.10 | 0.23 |
|  | *HLA-A*23- (Bw4A)* | 0.978(7748/7923) | 0.978(11565/11828) | 0.99 | 0.10 | 0.96 |  | 0.982(3371/3433) | 0.985(14134/14342) | 0.81 | 0.15 | 0.14 |  |
| *2DL3+* | *HLA-C*08+ (C1)* | 0.946(385/407) | 0.92(894/972) | 1.67 | 0.25 | **0.04** | **0.04** | 0.931(161/173) | 0.94(989/1052) | 0.81 | 0.33 | 0.53 | 0.68 |
|  | *HLA-C*08- (C1)* | 0.927(7135/7700) | 0.932(10479/11242) | 0.92 | 0.06 | 0.16 |  | 0.933(3101/3324) | 0.934(12879/13792) | 0.99 | 0.08 | 0.91 |  |
| *2DS2++* | *HLA-C*08+ (C1)* | 0.054(22/407) | 0.08(78/972) | 0.60 | 0.25 | **0.04** | **0.04** | 0.069(12/173) | 0.06(63/1052) | 1.23 | 0.33 | 0.53 | 0.64 |
|  | *HLA-C*08- (C1)* | 0.073(563/7700) | 0.068(763/11242) | 1.08 | 0.06 | 0.18 |  | 0.066(220/3324) | 0.067(918/13792) | 0.99 | 0.08 | 0.88 |  |
| *3DS1++* | *HLA-A*26+* | 0.068(39/575) | 0.046(25/548) | 1.51 | 0.27 | 0.12 | **0.04** | 0.053(12/228) | 0.043(26/606) | 1.26 | 0.36 | 0.53 | 0.45 |
|  | *HLA-A*26-* | 0.037(281/7532) | 0.043(503/11666) | 0.86 | 0.08 | **0.04** |  | 0.037(120/3269) | 0.04(568/14238) | 0.92 | 0.10 | 0.40 |  |
| *2DL2++* | *HLA-A*02+* | 0.075(370/4933) | 0.065(398/6134) | 1.17 | 0.08 | **0.04** | **0.04** | 0.074(157/2129) | 0.067(494/7407) | 1.11 | 0.10 | 0.28 | 0.14 |
|  | *HLA-A*02-* | 0.068(217/3174) | 0.073(443/6080) | 0.92 | 0.09 | 0.34 |  | 0.058(79/1368) | 0.065(481/7437) | 0.88 | 0.13 | 0.30 |  |
| *2DS2++* | *HLA-A*02+* | 0.075(368/4933) | 0.065(398/6134) | 1.16 | 0.08 | **0.05** | **0.05** | 0.073(155/2129) | 0.067(494/7407) | 1.09 | 0.10 | 0.36 | 0.10 |
|  | *HLA-A*02-* | 0.068(217/3174) | 0.073(443/6080) | 0.92 | 0.09 | 0.34 |  | 0.056(77/1368) | 0.065(487/7437) | 0.84 | 0.13 | 0.18 |  |
| *2DL3+* | *HLA-A*02+* | 0.925(4564/4933) | 0.935(5736/6134) | 0.86 | 0.08 | **0.04** | **0.05** | 0.927(1973/2129) | 0.933(6913/7407) | 0.91 | 0.10 | 0.32 | 0.15 |
|  | *HLA-A*02-* | 0.931(2956/3174) | 0.927(5637/6080) | 1.08 | 0.09 | 0.37 |  | 0.942(1289/1368) | 0.935(6955/7437) | 1.14 | 0.13 | 0.29 |  |
| *2DS4W+* | *HLA-A*33+* | 0.338(46/136) | 0.458(104/227) | 0.61 | 0.23 | **0.03** | **0.04** | 0.36(18/50) | 0.398(86/216) | 0.85 | 0.34 | 0.63 | 0.67 |
|  | *HLA-A*33-* | 0.385(3066/7971) | 0.393(4708/11987) | 0.97 | 0.03 | 0.30 |  | 0.404(1392/3447) | 0.411(6012/14628) | 0.97 | 0.04 | 0.43 |  |
| *2DL2+* | *HLA-A*25+ (Bw4A)* | 0.393(84/214) | 0.483(231/478) | 0.68 | 0.17 | **0.02** | **0.04** | 0.372(32/86) | 0.464(268/577) | 0.65 | 0.25 | 0.08 | 0.13 |
|  | *HLA-A*25- (Bw4A)* | 0.456(3597/7893) | 0.462(5423/11736) | 0.97 | 0.03 | 0.34 |  | 0.437(1489/3411) | 0.443(6317/14267) | 0.97 | 0.04 | 0.48 |  |
| *2DS2+* | *HLA-A*25+ (Bw4A)* | 0.393(84/214) | 0.483(231/478) | 0.68 | 0.17 | **0.02** | **0.04** | 0.372(32/86) | 0.464(268/577) | 0.65 | 0.25 | 0.08 | 0.14 |
|  | *HLA-A*25- (Bw4A)* | 0.455(3595/7893) | 0.462(5423/11736) | 0.97 | 0.03 | 0.32 |  | 0.437(1491/3411) | 0.444(6328/14267) | 0.97 | 0.04 | 0.47 |  |
| *2DL3++* | *HLA-A*25+ (Bw4A)* | 0.607(130/214) | 0.517(247/478) | 1.48 | 0.17 | **0.02** | **0.04** | 0.628(54/86) | 0.536(309/577) | 1.53 | 0.25 | 0.08 | 0.14 |
|  | *HLA-A*25- (Bw4A)* | 0.545(4299/7893) | 0.538(6314/11736) | 1.03 | 0.03 | 0.32 |  | 0.563(1921/3411) | 0.557(7940/14267) | 1.03 | 0.04 | 0.46 |  |
| *2DS4D+* | *HLA-B*13+ (Bw4BT80)* | 0.892(174/195) | 0.828(379/458) | 1.74 | 0.27 | **0.04** | **0.04** | 0.899(62/69) | 0.807(461/571) | 2.23 | 0.42 | 0.06 | 0.09 |
|  | *HLA-B*13- (Bw4BT80)* | 0.835(6609/7912) | 0.831(9771/11756) | 1.03 | 0.04 | 0.42 |  | 0.827(2835/3428) | 0.82(11699/14273) | 1.05 | 0.05 | 0.33 |  |
