# Supplementary figures and images for: Epistatic interactions between killer immunoglobulin-like receptors and human leukocyte antigen ligands are associated with ankylosing spondylitis
Source: PLoS Genet. 2020 Aug 17;16(8):e1008906. doi: 10.1371/journal.pgen.1008906 (PMC7451988; doi:10.1371/journal.pgen.1008906)

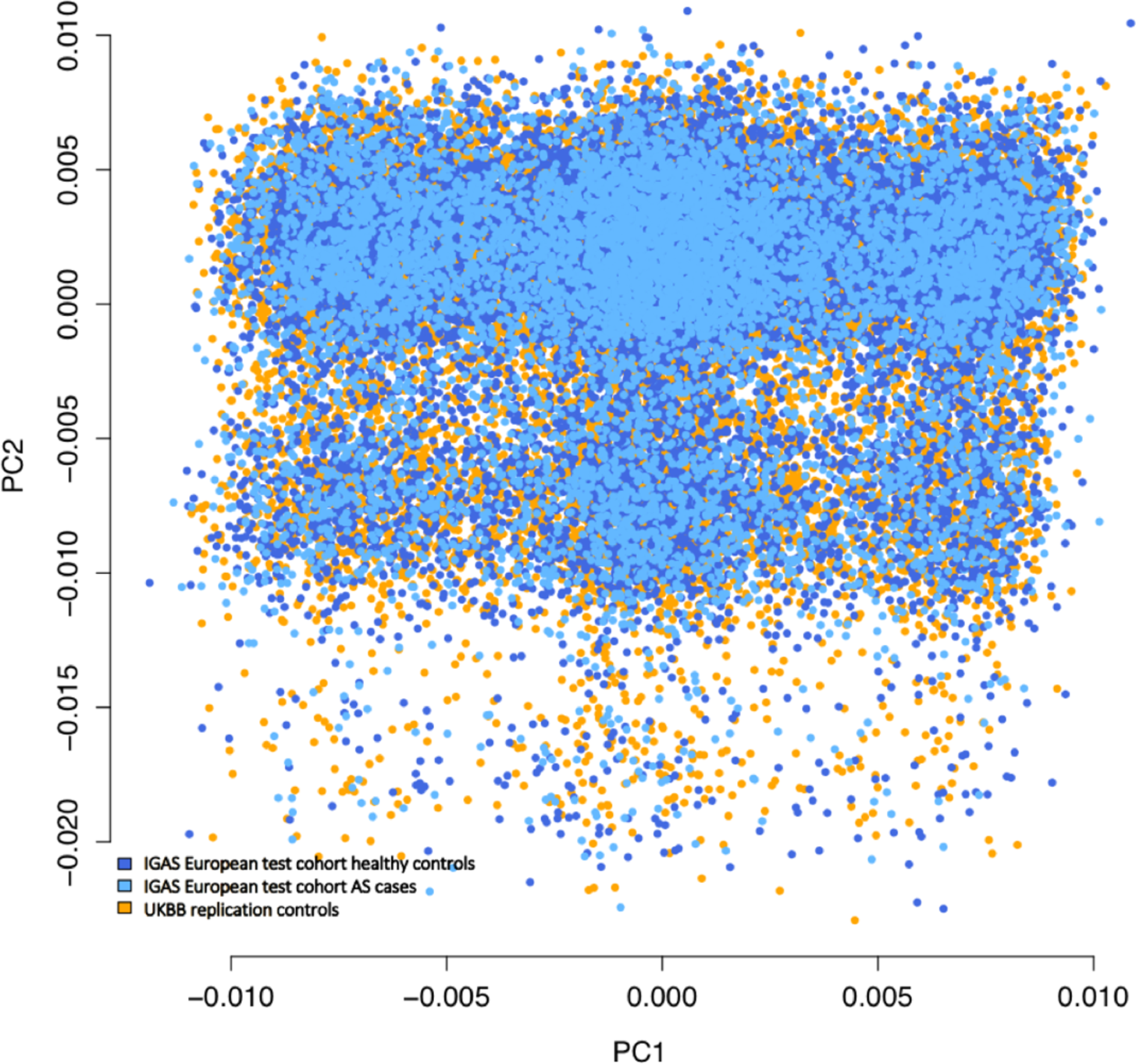

Supplement: S1 Fig — (TIF) [file pgen.1008906.s010.tif]

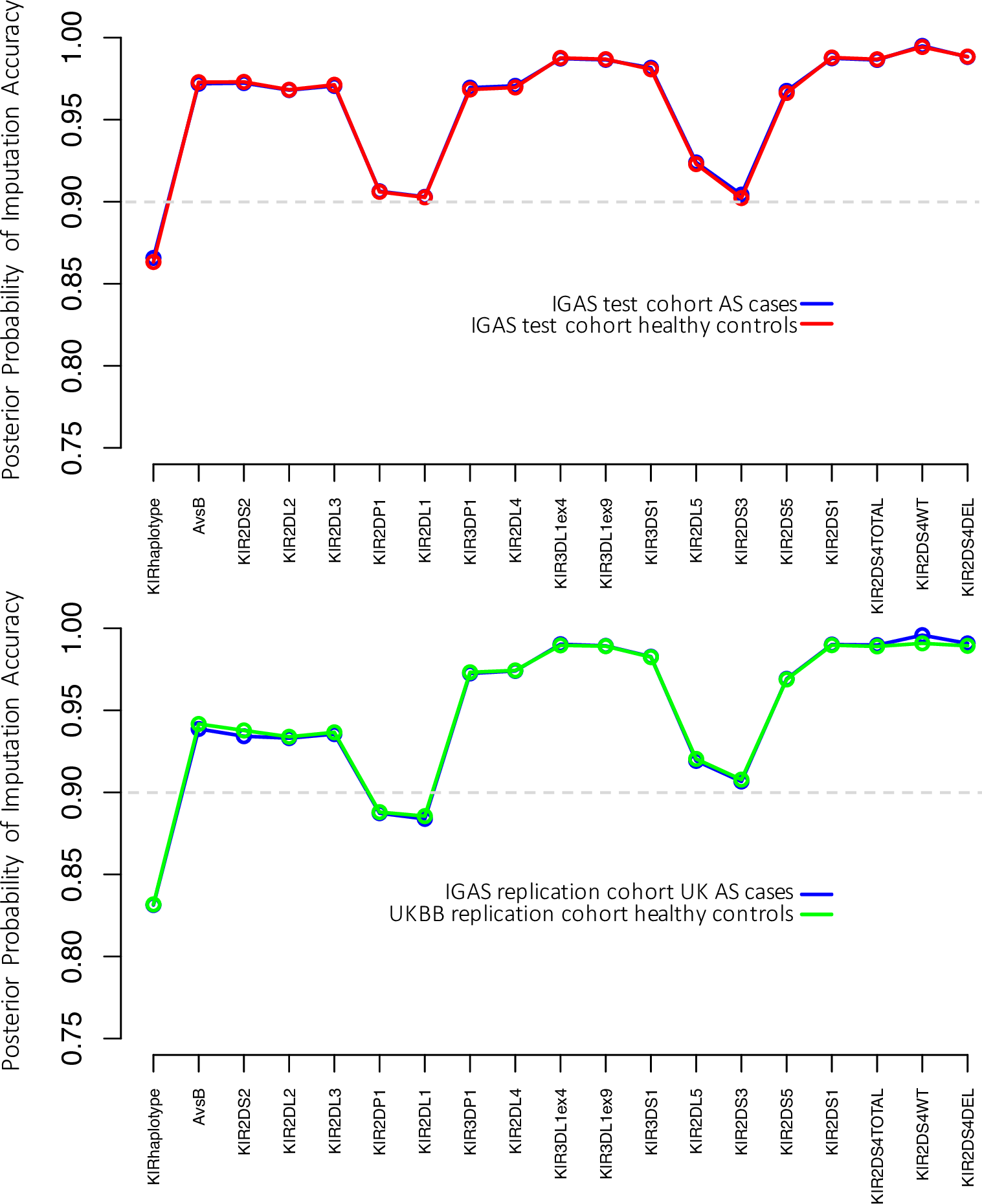

Supplement: S2 Fig — (TIF) [file pgen.1008906.s011.tif]

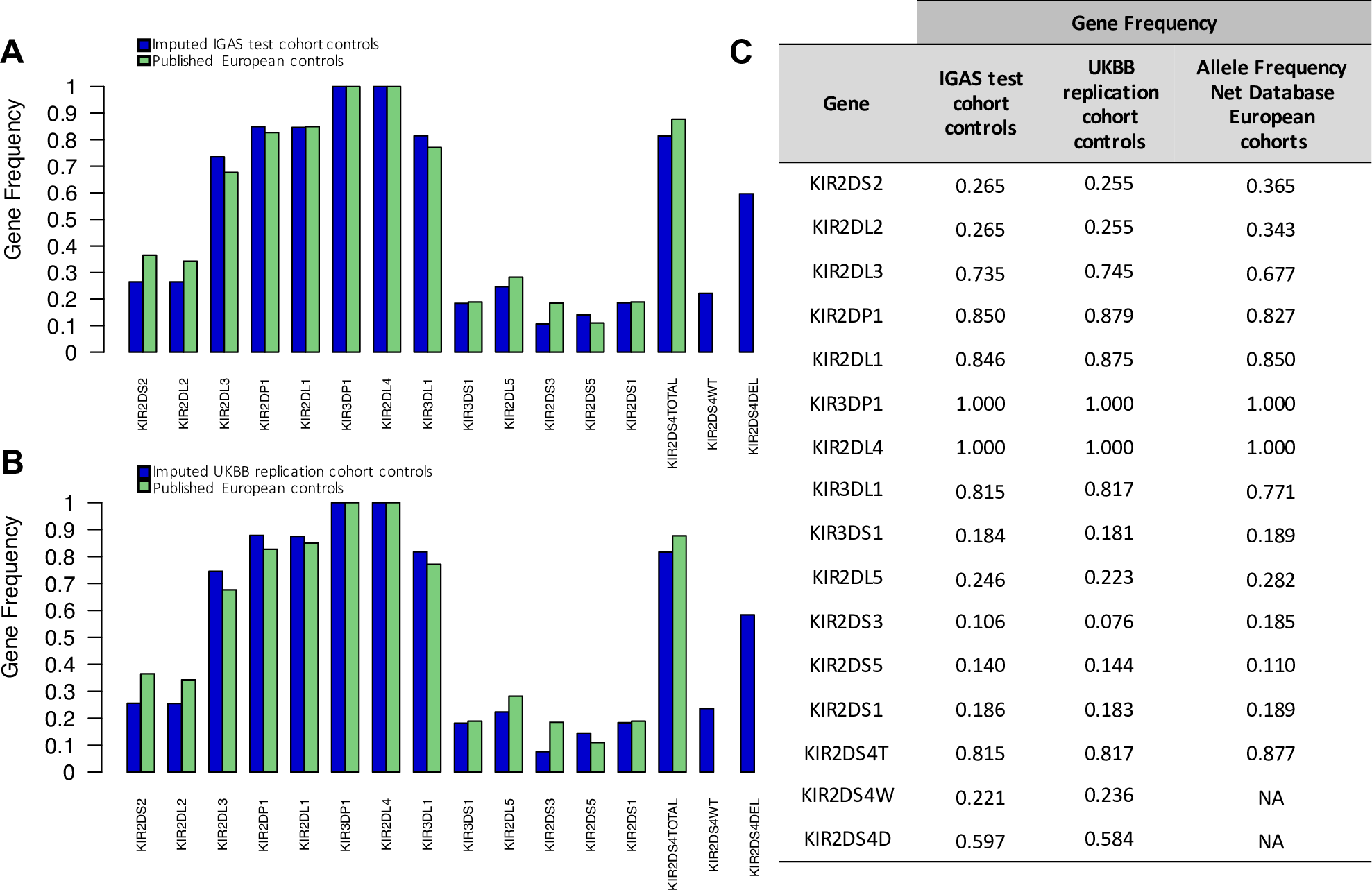

Supplement: S3 Fig — Bar charts show gene frequency comparisons for the test (A) and replication (B) cohorts with values enumerated in (C). KIR2DL5, KIR3DS3 and KIR2DS5 genes are duplicated on some B haplotypes and can occur on both the centromeric and telomeric halves of the haplotype. At present KIR*IMP is unable to distinguish centromeric from telomeric copies of these genes so they have been grouped together as a single locus. The database did not include frequencies for wild type and deletion variants of KIR2DS4. (TIF) [file pgen.1008906.s012.tif]

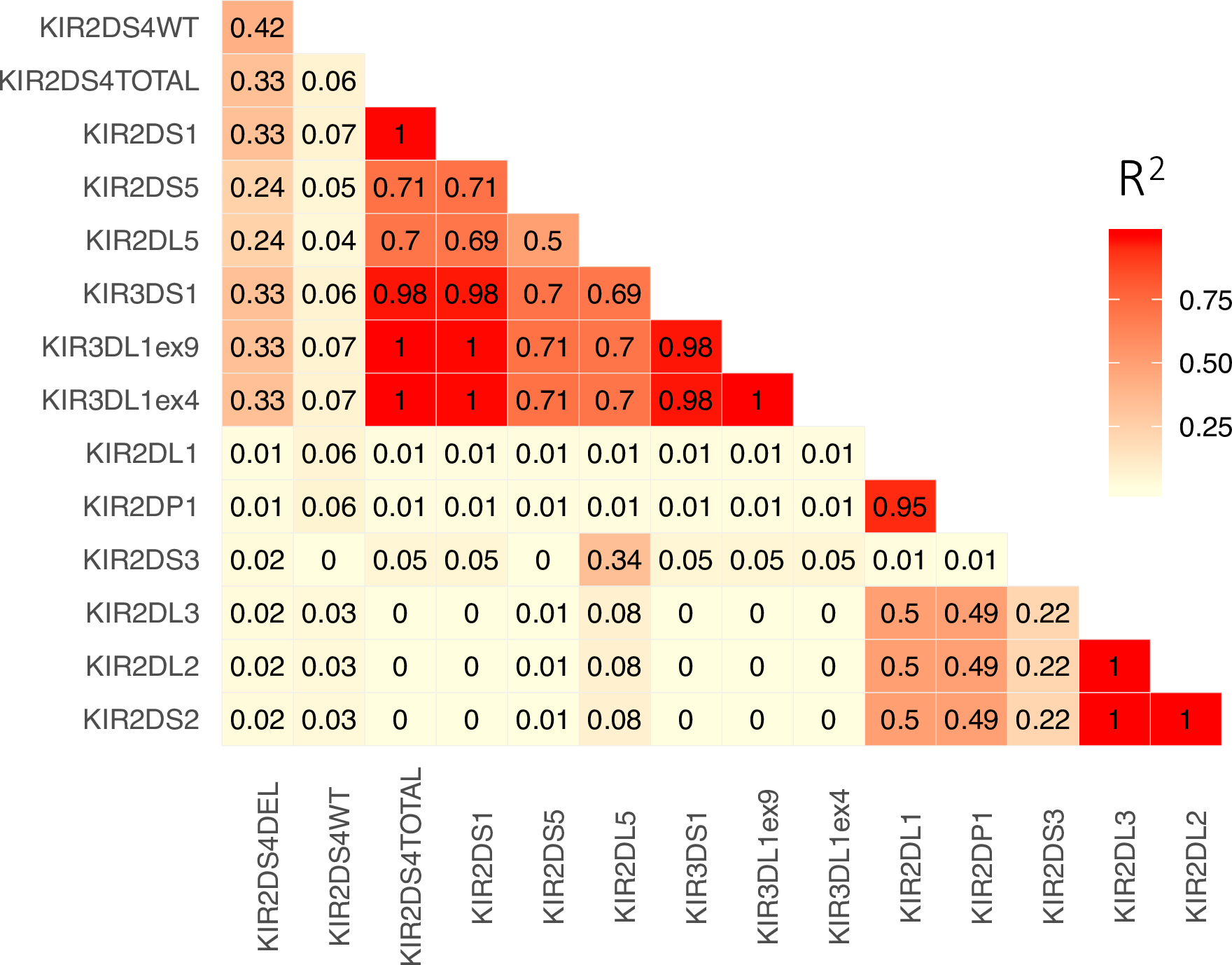

Supplement: S4 Fig — Strength of LD is coloured according to R2, with gene pairs in perfect positive or negative linkage (always or never occurring together in a haplotype) coloured red with an R2 value of 1. Genes are ordered according to genomic position from KIR2DS2 (centromeric) to KIR2DS4 (telomeric), with exclusion of framework genes KIR3DL3, KIR3DP1, KIR2DL4 and KIR3DL2. Distinction could not be made between centromeric and telomeric copies of KIR2DS3/5 or KIR2DL5. (TIF) [file pgen.1008906.s013.tif]
