# Supplementary material for: Epistatic interactions between killer immunoglobulin-like receptors and human leukocyte antigen ligands are associated with ankylosing spondylitis
Source: PLoS Genet. 2020 Aug 17;16(8):e1008906. doi: 10.1371/journal.pgen.1008906 (PMC7451988; doi:10.1371/journal.pgen.1008906)
Supplement: Attachment — Submitted filename: AS hla KIR 2020 0214 plos gene.docx [file pgen.1008906.s014.docx]

This is the largest association analysis of *KIR-HLA* to investigate the contribution of these complex receptors to AS immune-pathogenesis. However, nominally significant epistatic interactions between the genes encoding *KIR*s and *HLA* ligands were identified. Also, they exclude the three-way interaction *KIR3D* background modify the *HLA-B*27-ERAP1.* The exact effect of KIR gene and pro-inflammatory capacity of KIR expressing lymphocytes remain elusive.

Comment:

1. Authors made a lot of *KIR-HLA* review that decipher the field of possible mechanisms. No significant confirm functional experiments were accomplished.
2. They identified several *KIR2D/HLA-C1/C2* allele interactions beyond *HLA*-*B27*. As authors state a statistically interacting receptor-ligand pair may not biologically interact but rather tag closely linked genes. The effects of combinations interaction interpretation must be carefully.
3. What are the imputation weakness of HLA and KIR typing to gain accuracy since replication cohort show no significant findings?
4. What are the ethnic background difference?
