# Supplementary material for: Epistatic interactions between killer immunoglobulin-like receptors and human leukocyte antigen ligands are associated with ankylosing spondylitis
Source: PLoS Genet. 2020 Aug 17;16(8):e1008906. doi: 10.1371/journal.pgen.1008906 (PMC7451988; doi:10.1371/journal.pgen.1008906)
Supplement: Attachment — Submitted filename: ResponsetoReviewers_PLOSGenetics_KIRsinAS.docx [file pgen.1008906.s015.docx]

| **Editor/Reviewer Comments** | **Response** | **Revised text** | **Text location (page/paragraph)** |
| --- | --- | --- | --- |
| **Reviewer 1** | | | |
| 1. My first concern relates to the identification of healthy controls in the UK biobank cohort. Depending on the age of the individuals in this cohort, it might be difficult to be sure that they won’t develop AS later, especially if they are B27 positive. It would have been interesting to include only patient of >40 years old. | This is a valid concern given that ~5% of individuals who inherit the HLA-B*27 allele will go on to develop AS; this could potentially have a minor effect, reducing study power, but would not cause false positives. However, as the reviewer suggests, AS typically presents prior to 40 years of age, and all of the 500,000 individuals recruited for the UK Biobank public resource were aged between 40 and 69 years at the time of data collection. Although there may be some undocumented late-onset AS patients included in our UKB control cohort, this number is expected to be very small and unlikely to skew the results of statistical analyses.  We have included mention of the age of UKB controls in the Materials and Methods to avoid confusion. | “UKB controls, aged between 40-69 years, were selected through exclusion of individuals coding as having one of the following disease states: ankylosing spondylitis (code 1313), inflammatory bowel disease (code 1461), Crohn’s disease (code 1462), ulcerative colitis (code 1463), psoriasis (code 1453) or spine arthritis/spondylitis (code 1311)…” | Pg. 9, Pgph. 2 (Materials and Methods) |
| 2. Regarding KIR imputation: individuals with an imputed KIR haplotype posterior probability score <0.4 were excluded. The supplementary table 4 shows a perfect concordance between KIR imputed gene dosages and direct typing for a score >0.6. Why don’t you choose this threshold to increase the reliability of the imputation? | Although it would have been ideal to only include individuals for which KIR haplotypes were imputed with upmost accuracy in this study, in reality we were faced with a trade-off between overall imputation accuracy and sample size for statistical power. Increasing the KIR haplotype posterior probability threshold from 0.4 to 0.6 excluded >5000 additional individuals from analyses. Notably, in nearly all instances in which a haplotype was assigned a poor posterior probability score, it was only one or two of a few genes that were poorly imputed (namely KIR2DP1, KIR2DL1, KIR2DL5 or KIR2DS3 which are not as well typed by KIR*IMP as seen in Supp. Fig. 1 and Supp Table 4). Excluding large numbers of individuals based on the reduced imputation performance of these few genes seemed counter-productive in testing for genetic associations and epistatic interactions with those KIRs that were very well imputed, with the maximum possible statistical power.  Of note, many iterations of these statistical tests were conducted with varying stringencies of imputation quality control, including setting haplotype posterior probability thresholds of >0.8, >0.6, >0.4 and >0, and filtering samples based on per-gene imputation accuracy for each gene-specific test. The latter approach was considered undesirable as it meant that differing numbers of individuals were included in each association test, making findings across genes incomparable. Changing the KIR haplotype posterior probability threshold did not change the top ranked findings substantially for those KIR-HLA pairings in which large numbers of individuals were represented by each group, however the smaller cohort sizes resulting from higher stringency QC made rarer combinations of KIRs and HLA alleles less amenable to testing.  The threshold chosen was considered the best balance of imputation accuracy and sample size. Notably, in the publication accompanying the HLA imputation package HLA*IMP:03, the authors formally assessed the trade-off between imputation call threshold and study power in tests of genetic association. The effect of call threshold on sample size ratio (taken as the square of the Pearson correlation coefficient between true and imputed genotypes) was shown to be small, the modest improvement in imputation accuracy that resulted from an increased call threshold not sufficient to compensate for decreased call rate, such that the optimal threshold was 0. It would be possible to do a similar comparison for KIR*IMP imputed KIR dosages if a large cohort of both KIR typed and imputed individuals were available.  An additional statement has been added to the methods explaining the consequences of the chosen posterior probability threshold, and the limitations of the study more clearly outlined in the discussion. | For both cohorts, individuals with an imputed ‘*KIRhaplotype’* posterior probability score <0.4 were excluded, largely removing those haplotypes with poor imputation of genes *KIR2DP1, KIR2DL1, KIR2DL5* or *KIR2DS3,* which were the least well typed from available genotype data by the KIR*IMP algorithm.  “ *In silico* prediction of *KIR* dosages from genotype data has opened avenues for *KIR* association testing in cohorts too large for conventional laboratory-based typing methods. Albeit, prediction algorithms inherently operate with some degree of error, and the findings of computational studies should be validated with laboratory methods where possible.” | Pg. 11, Pgph. 2 (Materials and Methods)  Pg. 29, Pgph. 2 (Discussion) |
| 3. The multiple testing correction applied on the replication cohort should be explained in more details: have all the KIR/HLA interactions been tested only interactions with a significant pvalue in the Discovery cohort? | The multiple testing corrections applied to interaction P-values for each cohort were applied across the full complement of gene-gene interactions tested in that cohort (i.e. all interactions were tested in the replication cohort, not just those with a significant P-value in the test cohort).  We have made this clearer in the text. | “All analyses were conducted in both test and replication cohorts, however replication cohort results are shown only for the top gene associations and epistatic interactions returned from the test cohort. Where applied, correction for multiple testing was performed using the Bonferroni method with statistical significance defined as analyses achieving P<0.05. For interaction P-values, multiple testing correction was applied across all tested KIR-HLA interactions, for KIR association P-values, multiple testing correction was applied across all tested KIRs within the specified HLA subtype or allele group.” | Pg. 13, Pgph. 2 (Materials and Methods) |
| 4. It would be interesting to give the power of this study to detect epistatic interactions. | It is difficult to quote the power of this study to detect epistatic interactions overall. Statistical power would differ across each gene pair based on both KIR gene frequency and HLA subtype/allele frequency, which determines how often both constituents of the interaction are observed in combination. Providing a meaningful estimate of study power would require making many assumptions, including the strength of genetic epistasis between relevant KIR and HLA loci, for which there is presently no supporting literature available. It was expected that some gene-gene interactions would be detected in this study, given prior knowledge of the direct biological interactions that link KIRs and HLA ligands in an immunological context. More accurate estimation of the magnitude of these interaction effects on disease risk will require interrogation of larger, independent cohorts. |  |  |
| 5. The corrected p-values should be given in the table (and not only an asterisk indicating which p-values are <0.05 after correction). | Corrected P-values have been added where required in Tables 3 and 4. In all tables, ‘NS’ denoting (not significant) has also been replaced with the returned P-value |  | Pg. 17 (Table 3)  Pg. 19 (Table 4) |
| **Reviewer 2** | | | |
| 1. Authors made a lot of KIR-HLA review that decipher the field of possible mechanisms. No significant confirm functional experiments were accomplished. | This work was intended to be an exploratory statistical interrogation of KIR-HLA interactions in a large cohort. It is hoped that it will guide further functional studies addressing the role of specific KIR-HLA pairs in AS, or in immune responses more broadly. Notably, many previous publications have taken a computational approach to investigating the role of KIRs in disease, due to the complexity of the locus and the necessity of sizeable cohorts to identify meaningful trends (e.g. PMID: 32184413, PMID:31849952, PMID: 32102404)  Functional interrogation of lead findings was beyond the scope of this work and would require careful design of experimental approaches to assess and compare attributes of disease-risk associated and disease-protective KIR-HLA pairs (addressing features such as KIR/HLA cell surface expression, strength of receptor-ligand interactions, productive KIR signalling potential, the influence of HLA bound peptides, the influence of co-expressed competing KIRs etc.). We believe such work constitutes a number of independent studies. |  |  |
| 2. They identified several KIR2D/HLA-C1/C2 allele interactions beyond HLA-B27. As authors state a statistically interacting receptor-ligand pair may not biologically interact but rather tag closely linked genes. The effects of combinations interaction interpretation must be carefully. | We concur with the reviewer that the strong LD that exists across the KIR locus makes the interpretation of findings difficult, and have considered and commented on this is the text. The LD structure of the KIR locus and is shown explicitly in Supplementary Figure 3 (now Supplementary Figure 4). As the reviewer has quoted, we state in the discussion that *“given strong LD across both the HLA and KIR locus, members of a statistically interacting receptor-ligand pair may not biologically interact but rather tag closely linked genes for proteins that do engage to alter immune cell function”*. For this reason, the tables given in the text, showing top associated KIR-HLA interactions between receptors and ligands with functional evidence of productive engagement in the scientific literature, are expanded in the supplementary data to include all further interactions of the same significance between genes in LD. It is hoped all such data may be referenced in the future as understanding of KIR binding partners broaden.  The KIR-HLA interactions we comment upon in the discussion are those for which there is literature suggesting relevance to AS or other immune related conditions, and/or thorough functional studies demonstrating an immunological consequence of KIR-HLA engagement. We can only speculate on whether such interactions do in fact play a role in disease. We have added text to the discussion to make the limitations of this study clearer, emphasising that much work needs to be done to understand the relevance of our findings to disease immunopathogenesis. | “This study presents the first attempt to test for associations of *KIR-HLA* epistasis with an immune-mediated disease across all represented gene-gene combinations. Despite the substantial sample size of our test cohort, lack of an equally well powered replication cohort of independent AS patients made validation of findings difficult. Further, given strong LD across both the *HLA* and *KIR* locus, members of a statistically interacting receptor-ligand pair may not biologically interact but rather tag closely linked genes for proteins that do engage to alter immune cell function. Rare KIR haplotypes, in which unique gene combinations are observed, were likely overrepresented in those discarded during imputation quality filtering, hindering our ability dissect strong LD structures within this data. Here, only those interactions that can be interpreted in terms of experimentally validated KIR-HLA engagements are discussed, though to ascertain the relevance of these findings to AS pathogenesis on a functional level will require further experimentation.” | Pg. 27, Pgph. 1 (Discussion) |
| 3. What are the imputation weakness of HLA and KIR typing to gain accuracy since replication cohort show no significant findings? | HLA imputation performed equally well in both test and replication cohorts and is a well-established and extensively used approach to obtaining accurate HLA allele types to four-digit resolution (<https://doi.org/10.1101/091009>). It is unlikely that HLA imputation inaccuracy was a substantial weakness of this study.  As stated in the discussion,  *“We have endeavoured to replicate findings in a semi-independent cohort, incorporating UKB controls, however the lower imputation accuracy (ascribed to reduced SNP coverage) and reduced size of this sample set has hindered this somewhat.”*  the loss of KIR imputation accuracy in the replication cohort was likely attributed to reduced SNP coverage across the KIR locus on the UKB Affymetrix verses Immunochip array. This meant that more replication cohort samples were excluded under the KIR haplotype posterior probability threshold applied for imputation accuracy filtering, particularly reducing power to detect HLA interactions with KIR genes seen exclusively on KIR B haplotypes, which are rarer, less well imputed and thus were over represented in excluded haplotypes.  It should be noted that for the majority of the top KIR-HLA subtype and KIR-HLA allele interactions reported in the test cohort, replication cohort odds ratios are in close concordance, or at least show the same direction of effect. Thus it is likely that the loss of statistical significance in the replication cohort can be largely attributed to its reduced size. We have added a sentence addressing this in the discussion. | “Despite the substantial sample size of our test cohort, lack of an equally well powered replication cohort of independent AS patients made validation of findings difficult.” | Pg. 27, Pgph. 1 (Discussion) |
| 4. What are the ethnic background difference? | Both cohorts were filtered to be of predominantly European ethnicity by principal component analysis of 20,783 (Test) and 12,485 (Replication) autosomal SNPs.  We have included an additional supplementary figure showing the principal components analysis plot of both European cohorts used in this study. | PCs were recalculated for the filtered European test and replication cohorts (**Supplementary Fig. 1** shows stratification of the European cluster along the first two principal components) and the first ten principal components fitted as covariates in all regression models. | Pg. 12 (Supplementary Figures) |

NOTE: Reviewer 2 disagrees that all data underlying the results presented in this manuscript have been provided. On the contrary, all analyses presented here can be replicated using the data provided in Supplementary Tables 5, 6 and 9, which were uploaded to PLOS Genetics with our initial manuscript submission. Because of their size, these tables were separate to the main supplementary tables and figures file ‘Manuscript_PLOSGenetics_SUPPFiguresandTables.docx’ and may not have been included in the document downloaded for review.
